# Predicted molecules and signaling pathways for regulating seizures in the hippocampus in lithium-pilocarpine induced acute epileptic rats: A proteomics study

**DOI:** 10.3389/fncel.2022.947732

**Published:** 2022-12-01

**Authors:** Peng Wang, Lu Yang, Rang Yang, Zhangping Chen, Xiaofan Ren, Fangjiao Wang, Yan Jiao, Yuxin Ding, Fengyuan Yang, Tao Sun, Huisheng Ma

**Affiliations:** ^1^Ningxia Key Laboratory of Cerebrocranial Diseases, College of Traditional Chinese Medicine, Ningxia Medical University, Yinchuan, China; ^2^Ningxia Key Laboratory of Cerebrocranial Diseases, Ningxia Medical University, Yinchuan, China; ^3^School of Clinical Medicine, Ningxia Medical University, Yinchuan, China; ^4^Ningxia Key Laboratory of Cerebrocranial Diseases, Department of Neurosurgery, General Hospital of Ningxia Medical University, Ningxia Medical University, Yinchuan, China; ^5^College of Traditional Chinese Medicine, Ningxia Medical University, Yinchuan, China

**Keywords:** acute epilepsy, hippocampus, proteomics study, seizures, signaling pathways

## Abstract

Seizures in rodent models that are induced by lithium-pilocarpine mimic human seizures in a highly isomorphic manner. The hippocampus is a brain region that generates and spreads seizures. In order to understand the early phases of seizure events occurring in the hippocampus, global protein expression levels in the hippocampus on day 1 and day 3 were analyzed in lithium-pilocarpine induced acute epileptic rat models using a tandem mass tag-based proteomic approach. Our results showed that differentially expressed proteins were likely to be enhanced rather than prohibited in modulating seizure activity on days 1 and 3 in lithium-pilocarpine induced seizure rats. The differentially regulated proteins differed on days 1 and 3 in the seizure rats, indicating that different molecules and pathways are involved in seizure events occurring from day 1 to day 3 following lithium-pilocarpine administration. In regard to subcellular distribution, the results suggest that post-seizure cellular function in the hippocampus is possibly regulated in a differential manner on seizure progression. Gene ontology annotation results showed that, on day 1 following lithium-pilocarpine administration, it is likely necessary to regulate macromolecular complex assembly, and cell death, while on day 3, it may be necessary to modulate protein metabolic process, cytoplasm, and protein binding. Protein metabolic process rather than macromolecular complex assembly and cell death were affected on day 3 following lithium-pilocarpine administration. The extracellular matrix, receptors, and the constitution of plasma membranes were altered most strongly in the development of seizure events. In a KEGG pathway enrichment cluster analysis, the signaling pathways identified were relevant to sustained angiogenesis and evading apoptosis, and complement and coagulation cascades. On day 3, pathways relevant to Huntington’s disease, and tumor necrosis factor signaling were most prevalent. These results suggest that seizure events occurring in day 1 modulate macromolecular complex assembly and cell death, and in day 3 modulate biological protein metabolic process. In summary, our study found limited evidence for ongoing seizure events in the hippocampus of lithium-pilocarpine induced animal models; nevertheless, evaluating the global differential expression of proteins and their impacts on bio-function may offer new perspectives for studying epileptogenesis in the future.

## Introduction

Epilepsy manifests as repeated transient seizures with longer interictal periods between seizures. The primary goal of epilepsy research is to understand the mechanisms of epileptogenesis and ictogenesis. In epilepsy disorders, the brain tends to generate seizures (Fisher et al., 2005). The pilocarpine-induced animal model is commonly used as an epileptic seizure model that mimics the human disease in a highly isomorphic manner (Turski et al., 1983a,b).

Seizures induced by pilocarpine possibly exert their effects through the muscarinic receptor to cause an imbalance between excitatory and inhibitory transmission (Hamilton et al., 1997; Priel and Albuquerque, 2002). The vital characteristics of the pilocarpine model include rapid induction of acute status epilepticus (SE), the presence of a latent period and spontaneous recurrent seizures (SRSs, chronic phase) (Leite et al., 1990; Cavalheiro et al., 1991), the occurrence of widespread lesions, and seizures that are poorly controlled by antiepileptic drugs (Glien et al., 2002; Chakir et al., 2006; André et al., 2007). In a modification of the pilocarpine model, pilocarpine has also been combined with lithium to achieve a reduction dose and increased sensitivity to pilocarpine for inducing seizures; this model is similar to the pilocarpine model behaviorally, metabolically, electrographically, and neuropathologically (Honchar et al., 1983; Clifford et al., 1987).

After injecting pilocarpine, ictal, and interictal epileptic events are evoked and a clear pattern of theta rhythms is evident in the hippocampus (Turski et al., 1983a,b). Along with seizure event development, electrographic seizures are originated in the hippocampus and are propagated from the hippocampus to the amygdala and neocortex (Turski et al., 1983a,b). However, these hippocampal alterations appear to intensify progressively until 80 days after SE. In view of the important role of the hippocampus in generating and spreading seizures in epilepsy, it is important to understand the mechanisms and molecule alterations during early seizure events in animal models and patients with epilepsy. Biochemical changes reflect critical alterations in integral processes during the development of seizure events, yet they have received limited attention. The proteome studies of the human hippocampus in patients with Alzheimer’s disease (Edgar et al., 1999b; Sultana et al., 2007; Begcevic et al., 2013; Hondius et al., 2016), non-CNS malignancies (Yang et al., 2004), and refractory temporal lobe epilepsy has been reported (Persike et al., 2012, 2018). The proteome studies of epileptic animal models in the chronic phase induced by the kindling and pilocarpine models have also studied (Sadeghi et al., 2021). However, studies of biochemical changes during seizures in the acute phase of epileptic animal models have been quite limited to date.

Given the known role of the hippocampus in seizure development, we examined molecules and signaling pathways that may plausibly regulate seizures in the hippocampus using tandem mass tag (TMT)-labeled quantitative proteomic analysis in a lithium-pilocarpine induced epileptic rat model. Our results show that differentially expressed proteins are likely to be enhanced rather than prohibited in modulating seizures in a lithium-pilocarpine induced rat model. On day 1 following lithium-pilocarpine administration, macromolecular complex assembly, RNA binding, the extracellular regulation, and cell death were mainly regulated in the hippocampus. On day 3 following lithium-pilocarpine administration, protein metabolic process, cytoplasm, and protein binding were generally modulated. Moreover, on day 1 following lithium-pilocarpine administration (compared with controls), the majority of regulated signaling pathways comprised pathways relevant to cancer (regulating sustained angiogenesis and evading apoptosis), and complement and coagulation cascades. On day 3 following lithium-pilocarpine administration (compared with controls), the majority of regulated signaling pathways were as follows: Huntington’s disease, tumor necrosis factor (TNF) signaling, tight junction, and nuclear factor (NF)-kappa B pathways. Our study may offer potential indicators for seizure development in the acute phase in epilepsy. Although our study found limited evidence for ongoing seizure events in the hippocampus of lithium-pilocarpine induced animal models, evaluating the global differential expression of proteins and their impacts on biological function is critical to understanding the features of seizure events and may offer new perspectives for studying epileptogenesis in the future.

## Materials and methods

### Lithium-pilocarpine induced status epilepticus rat

Epileptic seizure rats (male Sprague Dawley rats; weight, approximately 220 g, *n* = 3 in each experimental group) were induced by intraperitoneal (IP) injection of lithium (130 mg/kg in 0.9% saline)-pilocarpine hydrochloride (30 mg/kg in 0.9% saline, Sigma), as previously described (with minor modifications) (Wang et al., 2021). In the present study, only those animals whose convulsion activity reached scale IV and scale V activity levels (Racine, 1972) were utilized; convulsions were allowed to last for 30 min. Finally, convulsion activity was terminated using chloral hydrate (400 mg/kg, Damao, Tianjin, China). The mortality of epileptic seizure rat was 10%. In experiment, three animals were divided in control group, three survival epileptic seizure rats were terminated after 1 day; three survival epileptic seizure rats were terminated after 1 day.

Animals were housed with free access to food and water at 25°C for 1 and 3 days after lithium-pilocarpine administration. At the end of the study, the both hippocampus of each rat was collected for TMT-labeled quantitative proteomic analysis (Jingjie, Hangzhou, China). All protocols and procedures were approved by the National Institutes of Health and the ethics committee of Ningxia Medial University (Ningxia, China). We followed all relevant national and international guidelines for animal care and welfare (e.g., the ARRIVE guidelines) in conducting this study. Research involving animals and all protocols and procedures were approved by the National Institutes of Health and the animal welfare committee of Ningxia Medical University (Ethics Approval Number: 2019-151, Ningxia, China).

### Tandem mass tag-labeled quantitative proteomic analysis

The hippocampi obtained from epileptic rats from all experimental groups were analyzed by quantitative proteomic analysis. All collected samples were ground into cell powder using liquid nitrogen. Four volumes of lysis buffer (8 M urea, 1% Protease Inhibitor Cocktail) were added and the samples were sonicated three times on ice using a high intensity ultrasonic processor (Scientz, Ningbo, China). The supernatant was collected after centrifugation at 12,000 *g* at 4°C for 10 min, and protein concentrations were measured using a bicinchoninic acid assay kit according to the manufacturer’s instructions. After that, the supernatant was incubated with trypsin in order to digest the protein to a peptide product. The peptide was desalted using a Strata X C18 SPE column (Phenomenex, Torrance, CA, USA) and was vacuum-dried. The peptide was reconstituted in 0.5 M triethylammonium bicarbonate (TEAB) and labeled with a TMT kit according to the manufacturer’s protocol.

### Database search

The MaxQuant search engine (v.1.5.2.8^1^) was used to analyze the resulting tandem mass spectrometry (MS/MS) data, and the Human UniProt Database^2^ was concatenated with a reverse decoy database search for the tandem mass spectra. Trypsin/P was used as the cleavage enzyme, allowing for up to four missing cleavages. The set of mass tolerance for precursor ions in the first search was 20 ppm; this value was set to 5 ppm in the main search (0.02 da, mass tolerance for fragment ions). Carbamidomethyl on Cys was specified as a fixed modification, whereas acetylation and oxidation on Met were specified as variable modifications. The false discovery rate (FDR) was adjusted to <1% and the minimum score for modified peptides was set at >40.

### Gene ontology annotation

The UniProt-GOA database^3^ was used to perform Gene Ontology (GO) annotation of the proteome. Proteins were classified by Gene Ontology annotation based on three categories: biological processes, cellular components, and molecular function.

### Enrichment of gene ontology analysis

A two-tailed Fisher’s exact test was used to test the enrichment of the differentially expressed proteins against all identified proteins in each category of the GO annotation. A corrected *p*-value of <0.05 was considered statistically significant.

### Enrichment of pathway analysis

The Encyclopedia of Genes and Genomes (KEGG) database was used to identify enriched pathways using a two-tailed Fisher’s exact test for enrichment of differentially expressed proteins among all identified proteins. Pathways with a corrected *p*-value of <0.05 were considered statistically significant. These pathways were classified into hierarchical categories according to criteria applied within the KEGG website.

### Statistics

All data were reported as means ± standard errors of the mean (SEM) for the three independent experiments. Statistical analysis was performed with one-way analysis of variance (ANOVA), followed by Benjamini and Hochberg (BH) with FDR correction in code of R followed by a Tukey’s post-test (Jingjie, Hangzhou, China). Two-tailed Fisher’s exact tests were used to calculate the statistical significance of the values of the conditions in each comparison for each independent condition in GO analysis (UniProt-GOA database, see footnote 3, and the InterProScan soft) and KEGG (KEGG Orthology database, and KAAS). In all cases, the threshold for statistical significance was set at *p* < 0.05.

## Results

### Identification of differentially expressed proteins in the hippocampus

**FIGURE 1:**
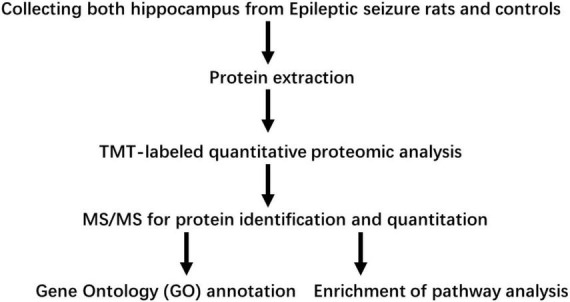
Workflow used in this study.

As shown in Figure 1, proteins in the hippocampus were analyzed in three biological replicates. To understand the early phase of seizure events occurring in the hippocampus, global protein expression levels in the hippocampus on day 1 and day 3 in lithium-pilocarpine induced acute epileptic rat models were analyzed using a TMT-based proteomic approach. In total, 6,157 proteins were identified, and 5,593 proteins were quantified. Therefore, the fold-change threshold was set to 1.2, and statistically significant values were defined as those with corrected *p*-values of <0.05.

On day 1 following lithium-pilocarpine administration, the expression of 89 proteins was upregulated, whereas the expression of 28 proteins was downregulated compared with controls (Table 1). On day 3 following lithium-pilocarpine administration, the expression of 34 proteins was promoted whereas that of 25 proteins was inhibited as compared with controls (Table 2).

**TABLE 1:** Differentially expression proteins on Day 1 comparing with control (ctrl) in hippocampus post ANOVA analysis.

Protein accession	Protein description	Gene name	Mean ± SEM (Day 1)	Mean ± SEM (Day 3)	Mean ± SEM (ctrl)	*P*-value (Day 1/Day 3/ctrl)	*P*-value (Day 1/ctrl)
A0A096MJE3	G1 to S phase transition 2	Gspt2	1.13 ± 0.073	0.946 ± 0.015	0.93 ± 0.104	0.037113703	0.044872317
A0A0A0MXY7	Uncharacterized protein	–	1.16 ± 0.031	1.038 ± 0.199	0.799 ± 0.042	0.025776779	0.023109735
A0A0A0MY39	ATP-binding cassette sub-family B member 9	Abcb9	1.246 ± 0.16	0.922 ± 0.042	0.839 ± 0.071	0.004760401	0.004728188
A0A0G2JY31	Alpha-1-antiproteinase	Serpina1	1.395 ± 0.324	0.868 ± 0.085	0.735 ± 0.048	0.004597287	0.004442122
A0A0G2JYD1	Ubiquitin-associated protein 2	Ubap2	1.082 ± 0.051	1.068 ± 0.061	0.854 ± 0.045	0.002524332	0.003693646
A0A0G2K2E2	Antizyme inhibitor 2	Azin2	1.092 ± 0.154	1.111 ± 0.018	0.804 ± 0.125	0.026624522	0.046602584
A0A0G2K5J5	Netrin G2	Ntng2	1.165 ± 0.13	0.991 ± 0.084	0.844 ± 0.057	0.013349057	0.010926122
A0A0G2K5T6	RNA polymerase I and III subunit C	Polr1c	1.159 ± 0.055	1.003 ± 0.135	0.837 ± 0.029	0.010943722	0.008969521
A0A0G2K624	Brain-derived neurotrophic factor	Bdnf	1.143 ± 0.151	1.08 ± 0.068	0.778 ± 0.089	0.008241653	0.009860647
A0A0G2K7W6	Similar to 60S ribosomal protein L27a	RGD1562402	1.075 ± 0.087	1.045 ± 0.127	0.838 ± 0.059	0.035376846	0.041811288
A0A0G2K9J8	LisH domain-containing protein ARMC9	Armc9	1.108 ± 0.163	1.033 ± 0.035	0.853 ± 0.061	0.041736227	0.04131062
A0A0G2QC03	Influenza virus NS1A-binding protein	Ivns1abp	1.123 ± 0.07	1.016 ± 0.076	0.861 ± 0.13	0.043382364	0.038141301
A0A0H2UHH9	40S ribosomal protein S24	Rps24	1.089 ± 0.039	1.068 ± 0.094	0.837 ± 0.113	0.027333621	0.035307812
A0A0H2UHI5	Serine protease inhibitor	Serpina3n	1.592 ± 0.268	0.795 ± 0.223	0.607 ± 0.066	0.002321611	0.002250448
A0A0H2UHP9	RCG39700, isoform CRA_d	Rab6a	1.158 ± 0.065	0.964 ± 0.067	0.881 ± 0.052	0.004523762	0.004012812
B1WBR8	F-box and leucine-rich repeat protein 4	Fbxl4	1.601 ± 0.104	0.684 ± 0.04	0.703 ± 0.049	0.0000058	0.0000113
B1WC40	Nuclear cap-binding protein subunit 2	Ncbp2	1.123 ± 0.015	0.947 ± 0.048	0.934 ± 0.039	0.001977601	0.002786505
B2GV14	Taxilin alpha OS = Rattus norvegicus	Txlna	1.112 ± 0.038	0.987 ± 0.05	0.903 ± 0.067	0.01015874	0.008415176
B2RYK2	2-(3-amino-3-carboxypropyl)histidine synthase subunit 1	Dph1	1.127 ± 0.123	0.978 ± 0.057	0.895 ± 0.048	0.03308807	0.028558263
B2RYW7	RCG26543, isoform CRA_b	Srp14	1.118 ± 0.121	0.973 ± 0.098	0.883 ± 0.025	0.046059745	0.039736226
D3ZB76	DnaJ (Hsp40) homolog, subfamily B, member 5 (Predicted)	Dnajb5	1.135 ± 0.088	0.994 ± 0.027	0.875 ± 0.012	0.001704428	0.001359745
D3ZBL6	Nucleoporin 160	Nup160	1.123 ± 0.107	1.071 ± 0.135	0.805 ± 0.115	0.034214132	0.039021308
D3ZGR7	RCG51149	Trir	1.185 ± 0.087	0.886 ± 0.048	0.932 ± 0.092	0.008574615	0.022044093
D3ZHV3	Metallothionein	Mt1m	1.378 ± 0.206	1.092 ± 0.433	0.47 ± 0.032	0.009031572	0.008623115
D3ZML3	Cyclin-dependent kinase 11B	Cdk11b	1.119 ± 0.056	0.979 ± 0.05	0.91 ± 0.028	0.003917502	0.003392973
D3ZMQ0	MGA, MAX dimerization protein	Mga	1.125 ± 0.108	0.964 ± 0.029	0.919 ± 0.046	0.021165579	0.021150701
D3ZR12	Syntrophin, gamma 2	Sntg2	1.097 ± 0.087	1.068 ± 0.081	0.834 ± 0.093	0.018011349	0.022843965
D3ZWS6	*N*(alpha)-acetyltransferase 30, NatC catalytic subunit	Naa30	1.08 ± 0.038	1.057 ± 0.079	0.862 ± 0.029	0.003276391	0.004270506
D3ZXL5	Nuclear cap-binding subunit 3	Ncbp3	1.124 ± 0.171	1.033 ± 0.107	0.848 ± 0.036	0.048754609	0.045718377
D3ZYS7	G3BP stress granule assembly factor 1	G3bp1	1.082 ± 0.037	1.002 ± 0.052	0.896 ± 0.048	0.00805689	0.006715078
D4A017	Transmembrane protein 87A	Tmem87a	1.13 ± 0.101	0.985 ± 0.112	0.883 ± 0.042	0.03823588	0.032409648
D4A0W1	ER membrane protein complex subunit 4	Emc4	1.13 ± 0.05	0.903 ± 0.115	0.911 ± 0.068	0.02995042	0.049835285
D4A1U7	Round spermatid basic protein 1	Rsbn1	1.737 ± 0.104	0.621 ± 0.037	0.627 ± 0.048	0.0000018	0.0000031
D4A563	Pseudopodium-enriched atypical kinase 1	Peak1	1.065 ± 0.123	1.035 ± 0.027	0.875 ± 0.041	0.036089869	0.04251408
F1LP80	Neurosecretory protein VGF	Vgf	1.266 ± 0.1	1.041 ± 0.14	0.691 ± 0.042	0.000845572	0.000742592
F1LR84	Neuronal pentraxin-2	Nptx2	1.134 ± 0.093	1.183 ± 0.374	0.687 ± 0.031	0.031945884	0.049143426
F1LST1	Fibronectin	Fn1	1.408 ± 0.031	0.861 ± 0.019	0.721 ± 0.017	0.0000001	0.0000001
F1LTU4	Ribosome assembly factor mrt4	Mrto4	1.175 ± 0.08	0.911 ± 0.126	0.911 ± 0.048	0.021122368	0.033445134
F1LZW6	Solute carrier family 25 member 13	Slc25a13	1.176 ± 0.103	0.897 ± 0.052	0.94 ± 0.018	0.003932235	0.011410437
F1M0A0	Anoctamin	Ano3	1.18 ± 0.159	0.997 ± 0.125	0.826 ± 0.115	0.048790639	0.041219969
F7EUK4	Kininogen-1	Kng1	1.535 ± 0.102	1.012 ± 0.424	0.44 ± 0.112	0.004142626	0.003524826
G3V6S8	Serine/arginine-rich splicing factor 6	Srsf6	1.079 ± 0.038	1.024 ± 0.051	0.898 ± 0.045	0.006340998	0.005933366
G3V734	2,4-dienoyl CoA reductase 1, mitochondrial, isoform CRA_a	Decr1	1.081 ± 0.053	1.026 ± 0.064	0.896 ± 0.024	0.008005358	0.007554546
G3V7K3	Ceruloplasmin	Cp	1.232 ± 0.189	0.981 ± 0.235	0.799 ± 0.023	0.056300625	0.048127174
G3V8D0	ST8 alpha-*N*-acetyl-neuraminide alpha-2,8-sialyltransferase 3	St8sia3	1.06 ± 0.068	1.089 ± 0.064	0.858 ± 0.071	0.010077793	0.021673603
G3V9W2	Tyrosine-protein kinase	Jak1	1.072 ± 0.047	1.043 ± 0.064	0.885 ± 0.056	0.013013008	0.015147659
M0R965	Uncharacterized protein	LOC685025	1.265 ± 0.203	0.902 ± 0.081	0.837 ± 0.046	0.007874934	0.008723589
O35532	Methylsterol monooxygenase 1	Msmo1	1.117 ± 0.046	0.982 ± 0.083	0.906 ± 0.036	0.012941343	0.011098348
O35547	Long-chain-fatty-acid–CoA ligase 4	Acsl4	1.108 ± 0.078	0.987 ± 0.047	0.919 ± 0.028	0.013900716	0.011886134
O35760	Isopentenyl-diphosphate Delta-isomerase 1	Idi1	1.104 ± 0.008	1.006 ± 0.074	0.912 ± 0.083	0.037623131	0.031500687
O35821	Myb-binding protein 1A	Mybbp1a	1.135 ± 0.07	0.994 ± 0.046	0.894 ± 0.047	0.005112057	0.004164782
P01048	T-kininogen 1	Map1	1.385 ± 0.381	1.059 ± 0.589	0.538 ± 0.079	0.043108294	0.037977515
P02680	Fibrinogen gamma chain	Fgg	1.561 ± 0.303	0.786 ± 0.03	0.648 ± 0.067	0.000335828	0.00034631
P02803	Metallothionein-1	Mt1	1.531 ± 0.134	0.905 ± 0.236	0.543 ± 0.069	0.001470308	0.001174416
P04961	Proliferating cell nuclear antigen	Pcna	1.232 ± 0.181	0.965 ± 0.161	0.779 ± 0.034	0.018447444	0.015287816
P05943	Protein S100-A10	S100a10	1.637 ± 0.473	0.698 ± 0.144	0.645 ± 0.186	0.008089812	0.010515729
P06238	Alpha-2-macroglobulin	A2m	1.361 ± 0.047	0.962 ± 0.147	0.666 ± 0.074	0.000792912	0.000628868
P06762	Heme oxygenase 1	Hmox1	1.687 ± 0.622	0.826 ± 0.107	0.491 ± 0.06	0.001963084	0.001591598
P14480	Fibrinogen beta chain	Fgb	1.598 ± 0.364	0.758 ± 0.021	0.66 ± 0.046	0.000529715	0.000620191
P16975	SPARC	Sparc	1.08 ± 0.057	1.053 ± 0.118	0.874 ± 0.033	0.025690482	0.030097449
P20059	Hemopexin	Hpx	1.409 ± 0.131	0.867 ± 0.12	0.708 ± 0.041	0.000397538	0.000368949
P30713	Glutathione S-transferase theta-2	Gstt2	1.146 ± 0.115	0.982 ± 0.026	0.875 ± 0.067	0.012206229	0.010099762
P35355	Prostaglandin G/H synthase 2	Ptgs2	1.493 ± 0.543	0.985 ± 0.213	0.526 ± 0.071	0.006710757	0.00566147
P52631	Signal transducer and activator of transcription 3	Stat3	1.085 ± 0.07	1.065 ± 0.172	0.815 ± 0.009	0.030420301	0.03815129
P59895	Serine/threonine-protein kinase Nek6	Nek6	1.179 ± 0.166	0.939 ± 0.102	0.887 ± 0.056	0.044020997	0.046634981
P61314	60S ribosomal protein L15	Rpl15	1.124 ± 0.058	1.076 ± 0.181	0.814 ± 0.095	0.039951166	0.044296005
P62912	60S ribosomal protein L32	Rpl32	1.087 ± 0.056	1.034 ± 0.107	0.875 ± 0.047	0.02843089	0.028255172
Q02765	Cathepsin S	Ctss	1.262 ± 0.097	0.884 ± 0.131	0.855 ± 0.162	0.027558651	0.034687309
Q3B8N7	TSC22 domain family protein 4	Tsc22d4	1.142 ± 0.127	1.005 ± 0.117	0.852 ± 0.059	0.036070482	0.030436262
Q3KR94	Vitronectin	Vtn	1.281 ± 0.201	0.832 ± 0.045	0.885 ± 0.074	0.006881687	0.016683615
Q3T1J1	Eukaryotic translation initiation factor 5A-1	Eif5a	1.288 ± 0.203	0.781 ± 0.069	0.952 ± 0.053	0.004786577	0.041311914
Q4FZZ3	Glutathione S-transferase alpha-5	Gsta5	1.898 ± 0.124	0.571 ± 0.047	0.51 ± 0.034	0.0000009	0.0000014
Q4KM45	UPF0687 protein C20orf27 homolog	–	1.086 ± 0.077	1.035 ± 0.092	0.864 ± 0.025	0.015008625	0.015455778
Q5HZA2	Sprouty RTK-signaling antagonist 2	Spry2	1.103 ± 0.117	1.059 ± 0.074	0.845 ± 0.079	0.025569912	0.029512024
Q5PPG2	Legumain	Lgmn	1.091 ± 0.074	1.091 ± 0.201	0.794 ± 0.054	0.035860633	0.049602943
Q5PPG5	Chga protein	Chga	1.208 ± 0.062	0.948 ± 0.079	0.845 ± 0.061	0.002155917	0.001929208
Q5U3Y8	Transcription factor BTF3	Btf3	1.108 ± 0.047	0.986 ± 0.043	0.91 ± 0.01	0.001514494	0.001244569
Q5XI28	Ribonucleoprotein PTB-binding 1	Raver1	1.114 ± 0.088	1.022 ± 0.092	0.87 ± 0.095	0.04198281	0.037441659
Q63041	Alpha-1-macroglobulin	A1m	1.162 ± 0.186	1.009 ± 0.067	0.86 ± 0.073	0.055049097	0.046839218
Q66HA8	Heat shock protein 105 kDa	Hsph1	1.087 ± 0.031	1.027 ± 0.089	0.885 ± 0.019	0.00900224	0.008450019
Q68FY4	Group specific component	Gc	1.316 ± 0.256	0.857 ± 0.014	0.833 ± 0.027	0.004232581	0.005987128
Q6P734	Plasma protease C1 inhibitor	Serping1	1.229 ± 0.027	1 ± 0.169	0.771 ± 0.026	0.003261921	0.002631873
Q6QI89	Mortality factor 4-like protein 2	Morf4l2	1.175 ± 0.135	1.017 ± 0.016	0.813 ± 0.058	0.003410756	0.002867195
Q71UF4	Histone-binding protein RBBP7	Rbbp7	1.109 ± 0.104	0.969 ± 0.038	0.905 ± 0.025	0.016424534	0.014568544
Q7TQ70	Ac1873	Fga	1.558 ± 0.316	0.767 ± 0.056	0.679 ± 0.072	0.000687567	0.000821385
Q9EPX0	Heat shock protein beta-8	Hspb8	1.167 ± 0.061	1.009 ± 0.109	0.826 ± 0.012	0.00311031	0.002534785
Q9EST6	Acidic leucine-rich nuclear phosphoprotein 32 family member B	Anp32b	1.124 ± 0.054	0.959 ± 0.052	0.924 ± 0.042	0.005966698	0.006510222
Q9QZK5	Serine protease HTRA1	Htra1	1.39 ± 0.485	0.916 ± 0.089	0.715 ± 0.012	0.02251602	0.01927177
Q9WVJ6	Tissue-type transglutaminase	Tgm2	1.204 ± 0.06	0.982 ± 0.172	0.826 ± 0.015	0.013910287	0.011555675
A0A0G2JX25	GMP reductase	Gmpr2	0.889 ± 0.106	0.987 ± 0.056	1.118 ± 0.047	0.035514647	0.02978354
A0A0G2K654	Histone cluster 1 H1 family member c	Hist1h1c	0.749 ± 0.085	1.017 ± 0.096	1.254 ± 0.21	0.00792144	0.006647937
A0A0G2K946	SPARC/osteonectin, cwcv and kazal-like domains proteoglycan 2	Spock2	0.924 ± 0.042	0.979 ± 0.013	1.114 ± 0.032	0.001052458	0.000947972
A0A0G2KA11	Phosphatidylinositol-3,4,5-trisphosphate-dependent Rac exchange factor 2	Prex2	0.855 ± 0.071	1.005 ± 0.093	1.111 ± 0.079	0.022575311	0.019371419
A0A0H2UHF5	ATP-sensitive inward rectifier potassium channel 10	Kcnj10	0.766 ± 0.01	0.985 ± 0.237	1.211 ± 0.13	0.027707889	0.023028924
A0A1W2Q674	Claudin	Cldn10	0.889 ± 0.057	1.014 ± 0.017	1.104 ± 0.084	0.011983829	0.010179599
B2RYI0	WD repeat-containing protein 91	Wdr91	0.899 ± 0.045	1.027 ± 0.023	1.084 ± 0.012	0.001055191	0.000976489
B5DF45	TNF receptor-associated factor 6	Traf6	0.928 ± 0.056	0.963 ± 0.054	1.119 ± 0.058	0.013804642	0.01466275
D3ZA21	Pleckstrin homology and RhoGEF domain-containing G3	Plekhg3	0.881 ± 0.129	1.002 ± 0.048	1.128 ± 0.021	0.03315667	0.027745196
D3ZBN0	Histone H1.5	Hist1h1b	0.86 ± 0.076	0.985 ± 0.056	1.17 ± 0.168	0.035167578	0.029578354
D3ZCB9	Family with sequence similarity 92, member B	Fam92b	0.913 ± 0.052	1.022 ± 0.041	1.112 ± 0.072	0.013409659	0.011172678
D3ZIF0	Zinc finger protein 512	Zfp512	0.688 ± 0.049	1.112 ± 0.331	1.208 ± 0.219	0.046057772	0.048803418
D3ZXL9	Potassium channel tetramerization domain-containing 4	Kctd4	0.931 ± 0.087	0.903 ± 0.032	1.172 ± 0.026	0.002745378	0.006217505
D3ZYJ5	GRAM domain-containing 1B	Gramd1b	0.943 ± 0.057	0.933 ± 0.087	1.136 ± 0.04	0.017315829	0.030984495
F1LPX0	Mitochondrial intermediate peptidase	Mipep	0.904 ± 0.01	0.992 ± 0.026	1.118 ± 0.038	0.000160503	0.000128185
F1LU97	SAM and SH3 domain-containing 1	Sash1	0.852 ± 0.064	0.989 ± 0.095	1.167 ± 0.158	0.031821982	0.026564789
F1LX28	Acyl-CoA thioesterase 11	Acot11	0.938 ± 0.062	0.917 ± 0.113	1.16 ± 0.029	0.024426638	0.047601114
F1M695	YjeF N-terminal domain-containing 3	Yjefn3	0.912 ± 0.041	0.988 ± 0.048	1.108 ± 0.04	0.004614964	0.003820617
F1M8H7	Actin-associated protein FAM107A	Fam107a	0.768 ± 0.057	1.146 ± 0.023	1.079 ± 0.026	0.0000772	0.000236951
G3V714	Neuroendocrine protein 7B2	Scg5	0.937 ± 0.052	0.982 ± 0.042	1.139 ± 0.074	0.011670151	0.011575582
G3V7Z4	Glia-derived nexin	Serpine2	0.812 ± 0.044	1.124 ± 0.077	1.09 ± 0.064	0.001105092	0.002519461
P08050	Gap junction alpha-1 protein	Gja1	0.945 ± 0.06	0.898 ± 0.033	1.183 ± 0.08	0.00251446	0.007650803
P43278	Histone H1.0	H1f0	0.794 ± 0.043	1.039 ± 0.248	1.201 ± 0.12	0.041964103	0.036372871
P62804	Histone H4	Hist1h4b	0.835 ± 0.053	1.053 ± 0.136	1.112 ± 0.108	0.031334673	0.032976035
Q4KLZ1	Transmembrane protein 186	Tmem186	0.917 ± 0.053	0.96 ± 0.096	1.133 ± 0.006	0.01949487	0.020474501
Q5XI90	Dynein light chain Tctex-type 3	Dynlt3	0.786 ± 0.171	0.844 ± 0.195	1.387 ± 0.145	0.024011807	0.029094976
Q63357	Unconventional myosin-Id	Myo1d	0.909 ± 0.062	1 ± 0.073	1.108 ± 0.087	0.042586877	0.035800118
Q9Z122	Acyl-CoA 6-desaturase	Fads2	0.791 ± 0.023	1.118 ± 0.129	1.106 ± 0.026	0.001449412	0.002601096

**TABLE 2:** Differentially expression proteins on Day 3 comparing with control (ctrl) in hippocampus post ANOVA analysis.

Protein accession	Protein description	Gene name	Mean ± SEM (Day 1)	Mean ± SEM (Day 3)	Mean ± SEM (ctrl)	*P*-value (Day 1/Day 3/ctrl)	*P*-value (Day 3/ctrl)
A0A0G2JWD6	AP-3 complex subunit beta	Ap3b1	1.045 ± 0.022	1.076 ± 0.097	0.881 ± 0.082	0.037565563	0.04375288
A0A0G2JYD1	Ubiquitin-associated protein 2	Ubap2	1.082 ± 0.051	1.068 ± 0.061	0.854 ± 0.045	0.002524332	0.00490896
A0A0G2K1W1	RAB11 family-interacting protein 5	Rab11fip5	1.054 ± 0.051	1.072 ± 0.066	0.885 ± 0.091	0.036543169	0.04601017
A0A0G2K2E2	Antizyme inhibitor 2	Azin2	1.092 ± 0.154	1.111 ± 0.018	0.804 ± 0.125	0.026624522	0.0349248
A0A0G2K624	Brain-derived neurotrophic factor	Bdnf	1.143 ± 0.151	1.08 ± 0.068	0.778 ± 0.089	0.008241653	0.01924202
A0A0G2K890	Ezrin	Ezr	0.988 ± 0.066	1.147 ± 0.197	0.853 ± 0.029	0.049277747	0.04156111
A0A0H2UHH9	40S ribosomal protein S24	Rps24	1.089 ± 0.039	1.068 ± 0.094	0.837 ± 0.113	0.027333621	0.04861763
B1WBV1	Axin interactor, dorsalization-associated	Aida	0.995 ± 0.067	1.117 ± 0.09	0.891 ± 0.064	0.027138702	0.02254102
D3ZB30	Polypyrimidine tract binding protein 1, isoform CRA_c	Ptbp1	1.029 ± 0.117	1.087 ± 0.049	0.893 ± 0.019	0.038545174	0.03592311
D3ZDM7	D-aspartate oxidase	Ddo	0.99 ± 0.075	1.108 ± 0.069	0.908 ± 0.08	0.048741759	0.04155139
D3ZHV3	Metallothionein	Mt1m	1.378 ± 0.206	1.092 ± 0.433	0.47 ± 0.032	0.009031572	0.03498570
D3ZR12	Syntrophin, gamma 2	Sntg2	1.097 ± 0.087	1.068 ± 0.081	0.834 ± 0.093	0.018011349	0.03484456
D3ZWS6	*N*(alpha)-acetyltransferase 30, NatC catalytic subunit	Naa30	1.08 ± 0.038	1.057 ± 0.079	0.862 ± 0.029	0.003276391	0.00730703
D4A8F2	Ras suppressor protein 1	Rsu1	0.99 ± 0.053	1.117 ± 0.107	0.9 ± 0.082	0.051994285	0.04422024
F1LP80	Neurosecretory protein VGF	Vgf	1.266 ± 0.1	1.041 ± 0.14	0.691 ± 0.042	0.000845572	0.0060229
F1LQ22	Unconventional SNARE in the ER 1	Use1	1.046 ± 0.054	1.082 ± 0.072	0.871 ± 0.109	0.040226676	0.04558807
F1LR84	Neuronal pentraxin-2	Nptx2	1.134 ± 0.093	1.183 ± 0.374	0.687 ± 0.031	0.031945884	0.04578122
F1LS29	Calpain-1 catalytic subunit	Capn1	0.999 ± 0.083	1.103 ± 0.06	0.902 ± 0.064	0.035158138	0.02937753
F1LVR8	Myocardin-related transcription factor A	Mrtfa	1.045 ± 0.114	1.076 ± 0.033	0.885 ± 0.037	0.032501038	0.03582711
F7EUK4	Kininogen-1	Kng1	1.535 ± 0.102	1.012 ± 0.424	0.44 ± 0.112	0.004142626	0.03027817
G3V8D0	ST8 alpha-*N*-acetyl-neuraminide alpha-2,8-sialyltransferase 3	St8sia3	1.06 ± 0.068	1.089 ± 0.064	0.858 ± 0.071	0.010077793	0.01250406
M0RC17	Cell adhesion molecule L1-like	Chl1	0.98 ± 0.036	1.115 ± 0.112	0.914 ± 0.066	0.045954776	0.04065344
O35263	Platelet-activating factor acetylhydrolase IB subunit gamma	Pafah1b3	1.003 ± 0.072	1.135 ± 0.141	0.886 ± 0.049	0.038926018	0.03261374
O35314	Secretogranin-1	Chgb	1.022 ± 0.02	1.093 ± 0.116	0.88 ± 0.043	0.021703401	0.02019491
P02803	Metallothionein-1	Mt1	1.531 ± 0.134	0.905 ± 0.236	0.543 ± 0.069	0.001470308	0.04114526
P06238	Alpha-2-macroglobulin	A2m	1.361 ± 0.047	0.962 ± 0.147	0.666 ± 0.074	0.000792912	0.01891073
P35355	Prostaglandin G/H synthase 2	Ptgs2	1.493 ± 0.543	0.985 ± 0.213	0.526 ± 0.071	0.006710757	0.04898848
P55063	Heat shock 70 kDa protein 1-like	Hspa1l	0.807 ± 0.104	1.472 ± 0.486	0.731 ± 0.082	0.024450167	0.02812714
Q4W1H3	Myosin 9b	Myo9b	0.982 ± 0.14	1.124 ± 0.046	0.895 ± 0.048	0.052509487	0.04591866
Q5XI44	X-ray repair complementing defective repair in Chinese hamster cells 4	Xrcc4	1.026 ± 0.027	1.085 ± 0.047	0.893 ± 0.049	0.004050963	0.003805
Q6P734	Plasma protease C1 inhibitor	Serping1	1.229 ± 0.027	1 ± 0.169	0.771 ± 0.026	0.003261921	0.04450734
Q6QI89	Mortality factor 4-like protein 2	Morf4l2	1.175 ± 0.135	1.017 ± 0.016	0.813 ± 0.058	0.003410756	0.02788628
Q925D4	Transmembrane protein 176B	Tmem176b	1.05 ± 0.205	1.242 ± 0.217	0.714 ± 0.188	0.045113157	0.04199842
Q9EPX0	Heat shock protein beta-8	Hspb8	1.167 ± 0.061	1.009 ± 0.109	0.826 ± 0.012	0.00311031	0.03531399
A0A0G2JZ56	Ankyrin 2	Ank2	0.966 ± 0.055	0.922 ± 0.048	1.126 ± 0.055	0.008529857	0.00860688
A0A0G2K1N9	Selenoprotein O	Selenoo	1.048 ± 0.065	0.868 ± 0.077	1.093 ± 0.077	0.018982576	0.02072012
A0A0G2K2R0	Uncharacterized protein	–	0.991 ± 0.047	0.897 ± 0.053	1.123 ± 0.046	0.004079903	0.00331745
A0A0G2K3 × 6	Uncharacterized protein	–	1.014 ± 0.071	0.857 ± 0.118	1.141 ± 0.097	0.036829653	0.03157403
A0A0G2K6H2	Maleylacetoacetate isomerase	Gstz1	1.013 ± 0.125	0.808 ± 0.107	1.146 ± 0.12	0.031248049	0.02763363
A0A0G2QC22	PAXX, non-homologous end joining factor	Paxx	0.973 ± 0.074	0.904 ± 0.043	1.14 ± 0.097	0.020130803	0.01832119
D3ZBN4	Ergosterol biosynthesis 28 homolog	Erg28	1.058 ± 0.123	0.863 ± 0.068	1.094 ± 0.078	0.037957654	0.04283949
D3ZXL9	Potassium channel tetramerization domain-containing 4	Kctd4	0.931 ± 0.087	0.903 ± 0.032	1.172 ± 0.026	0.002745378	0.00357546
D3ZYJ5	GRAM domain-containing 1B	Gramd1b	0.943 ± 0.057	0.933 ± 0.087	1.136 ± 0.04	0.017315829	0.02337596
D3ZZN3	Acetyl-coenzyme A synthetase	Acss1	1.014 ± 0.101	0.899 ± 0.068	1.094 ± 0.049	0.053259668	0.04616768
D4A7T8	Family with sequence similarity 81, member A	Fam81a	1.005 ± 0.04	0.885 ± 0.076	1.104 ± 0.11	0.041193021	0.03527131
F1LV07	Dynein, axonemal, heavy chain 9	Dnah9	0.973 ± 0.045	0.916 ± 0.067	1.122 ± 0.086	0.025205329	0.0232872
F1LX28	Acyl-CoA thioesterase 11	Acot11	0.938 ± 0.062	0.917 ± 0.113	1.16 ± 0.029	0.024426638	0.02991072
G3V7R4	Forkhead box protein O1	Foxo1	0.969 ± 0.086	0.906 ± 0.07	1.136 ± 0.101	0.044594782	0.0417731
G3V8F9	Alpha-methylacyl-CoA racemase	Amacr	1.096 ± 0.053	0.867 ± 0.054	1.048 ± 0.091	0.012544079	0.03458151
P08050	Gap junction alpha-1 protein	Gja1	0.945 ± 0.06	0.898 ± 0.033	1.183 ± 0.08	0.00251446	0.0027876
P11530	Dystrophin	Dmd	1.006 ± 0.089	0.875 ± 0.075	1.098 ± 0.043	0.025787682	0.02212798
P18484	AP-2 complex subunit alpha-2	Ap2a2	0.976 ± 0.035	0.888 ± 0.043	1.072 ± 0.069	0.011174105	0.00911871
P29534	Vascular cell adhesion protein 1	Vcam1	1.013 ± 0.036	0.891 ± 0.088	1.071 ± 0.019	0.022540728	0.02094853
P60825	Cold-inducible RNA-binding protein	Cirbp	1.006 ± 0.015	0.916 ± 0.054	1.113 ± 0.088	0.017995756	0.01481907
Q01984	Histamine *N*-methyltransferase	Hnmt	1.009 ± 0.091	0.822 ± 0.089	1.181 ± 0.001	0.00450368	0.00367635
Q3V5 × 8	Endonuclease G	Endog	0.962 ± 0.022	0.921 ± 0.093	1.128 ± 0.077	0.02816849	0.02842668
Q499N5	Acyl-CoA synthetase family member 2, mitochondrial	Acsf2	1.011 ± 0.034	0.888 ± 0.089	1.1 ± 0.043	0.017128151	0.01461175
Q5XI90	Dynein light chain Tctex-type 3	Dynlt3	0.786 ± 0.171	0.844 ± 0.195	1.387 ± 0.145	0.024011807	0.04765999
Q9EP88	Brain mitochondrial carrier protein BMCP1	Slc25a14	0.986 ± 0.085	0.898 ± 0.055	1.125 ± 0.117	0.051370739	0.04420038

These results indicate that protein expression is likely to be enhanced rather than prohibited in modulating seizures. Moreover, the expression levels of only 16 proteins were upregulated on both day 1 and day 3, while only five proteins were downregulated on both day 1 and day 3, suggesting that different molecules and pathways are involved in seizure events occurring from day 1 to day 3 following lithium-pilocarpine administration.

These results also suggest that the decreased number of upregulated proteins from day 1 to day 3 following lithium-pilocarpine administration may illustrate the possibility that the early phase of seizure events requires more molecules and activated pathways than are necessary in the late phase. Indeed, comparing day 3 to day 1 following lithium-pilocarpine administration, we found that 14 proteins were promoted and 37 proteins were impeded. Thus, only two upregulated proteins were the same on days 1 and 3 following lithium-pilocarpine administration (Table 3). These results suggest that epileptic events on day 1 and day 3 following lithium-pilocarpine administration require different molecules and different pathways for effective facilitation.

**TABLE 3:** Differentially expression proteins on Day 3 comparing with Day 1 in hippocampus post ANOVA analysis.

Protein accession	Protein description	Gene name	Mean ± SEM (Day 1)	Mean ± SEM (Day 3)	Mean ± SEM (ctrl)	*P*-value (Day 1/Day 3/ctrl)	*P*-value (Day 3/Day 1)
A0A0G2JVP4	Immunoglobulin heavy constant mu	Ighm	0.859 ± 0.071	1.182 ± 0.075	0.969 ± 0.102	0.011212146	0.00963031
A0A0U1RRP1	Synaptogyrin-1 (Fragment)	Syngr1	0.895 ± 0.062	1.085 ± 0.076	1.031 ± 0.063	0.030283802	0.02934154
D3ZA45	Autophagy-related protein 13	Atg13	0.91 ± 0.04	1.121 ± 0.071	0.975 ± 0.057	0.010603183	0.00946380
D3ZQN3	PNMA family member 8B	Pnma8b	0.887 ± 0.059	1.086 ± 0.084	1.034 ± 0.05	0.021802957	0.02188623
D4A469	Sestrin 3	Sesn3	0.872 ± 0.137	1.219 ± 0.119	0.919 ± 0.078	0.024385703	0.02685536
D4A542	G protein-coupled receptor-associated sorting protein 2	Gprasp2	0.893 ± 0.083	1.087 ± 0.052	1.03 ± 0.043	0.024300816	0.02332960
D4AAI8	Adhesion G protein-coupled receptor G7	Adgrg7	0.916 ± 0.046	1.108 ± 0.053	0.982 ± 0.059	0.012669651	0.01100500
F1M8H7	Actin-associated protein FAM107A	Fam107a	0.768 ± 0.057	1.146 ± 0.023	1.079 ± 0.026	0.0000772	0.0000932
G3V7Z4	Glia-derived nexin	Serpine2	0.812 ± 0.044	1.124 ± 0.077	1.09 ± 0.064	0.001105092	0.00149315
G3V917	Protein TANC1	Tanc1	0.922 ± 0.044	1.167 ± 0.16	0.941 ± 0.008	0.033418706	0.04178967
H9N1L3	BCL11B, BAF complex component	Bcl11b	0.9 ± 0.066	1.101 ± 0.058	1.005 ± 0.093	0.045468626	0.0383876
P97577	Fasciculation and elongation protein zeta-1	Fez1	0.877 ± 0.045	1.057 ± 0.073	1.015 ± 0.062	0.021340053	0.02207026
Q9R1K8	RAS guanyl-releasing protein 1	Rasgrp1	0.861 ± 0.097	1.145 ± 0.048	1.003 ± 0.111	0.02849426	0.02374808
Q9Z122	Acyl-CoA 6-desaturase	Fads2	0.791 ± 0.023	1.118 ± 0.129	1.106 ± 0.026	0.001449412	0.00234218
A0A0A0MY39	ATP-binding cassette sub-family B member 9	Abcb9	1.246 ± 0.16	0.922 ± 0.042	0.839 ± 0.071	0.004760401	0.01779094
A0A0G2JTL7	RBR-type E3 ubiquitin transferase	Ankib1	1.126 ± 0.104	0.893 ± 0.098	0.984 ± 0.067	0.054241554	0.04650385
A0A0G2JY31	Alpha-1-antiproteinase	Serpina1	1.395 ± 0.324	0.868 ± 0.085	0.735 ± 0.048	0.004597287	0.01874706
A0A0G2K1N9	Selenoprotein O	Selenoo	1.048 ± 0.065	0.868 ± 0.077	1.093 ± 0.077	0.018982576	0.04683435
A0A0H2UHI5	Serine protease inhibitor	Serpina3n	1.592 ± 0.268	0.795 ± 0.223	0.607 ± 0.066	0.002321611	0.01009321
A0A0H2UHP9	RCG39700, isoform CRA_d	Rab6a	1.158 ± 0.065	0.964 ± 0.067	0.881 ± 0.052	0.004523762	0.02576508
B1WBR8	F-box and leucine-rich repeat protein 4	Fbxl4	1.601 ± 0.104	0.684 ± 0.04	0.703 ± 0.049	0.0000058	0.0000093
D3Z899	Mitoguardin 2	Miga2	1.184 ± 0.136	0.82 ± 0.108	1.007 ± 0.085	0.017770309	0.01481953
D3ZGR7	RCG51149	Trir	1.185 ± 0.087	0.886 ± 0.048	0.932 ± 0.092	0.008574615	0.00969835
D4A0W1	ER membrane protein complex subunit 4	Emc4	1.13 ± 0.05	0.903 ± 0.115	0.911 ± 0.068	0.02995042	0.04036656
D4A1U7	Round spermatid basic protein 1	Rsbn1	1.737 ± 0.104	0.621 ± 0.037	0.627 ± 0.048	0.0000018	0.0000030
D4ABX8	Leucine-rich repeat and fibronectin Type-III domain-containing protein 4	Lrfn4	1.16 ± 0.076	0.832 ± 0.097	1.022 ± 0.15	0.035324956	0.03040222
F1LST1	Fibronectin	Fn1	1.408 ± 0.031	0.861 ± 0.019	0.721 ± 0.017	0.0000001	0.000001
F1LTD7	DENN domain-containing 4C	Dennd4c	1.096 ± 0.047	0.896 ± 0.105	1.014 ± 0.057	0.055019514	0.04824436
F1LTU4	Ribosome assembly factor mrt4	Mrto4	1.175 ± 0.08	0.911 ± 0.126	0.911 ± 0.048	0.021122368	0.03101664
F1LZW6	Solute carrier family 25 member 13	Slc25a13	1.176 ± 0.103	0.897 ± 0.052	0.94 ± 0.018	0.003932235	0.00437097
G3V8F9	Alpha-methylacyl-CoA racemase	Amacr	1.096 ± 0.053	0.867 ± 0.054	1.048 ± 0.091	0.012544079	0.01337644
M0R965	Uncharacterized protein	LOC685025	1.265 ± 0.203	0.902 ± 0.081	0.837 ± 0.046	0.007874934	0.02131333
P02680	Fibrinogen gamma chain	Fgg	1.561 ± 0.303	0.786 ± 0.03	0.648 ± 0.067	0.000335828	0.00139698
P02803	Metallothionein-1	Mt1	1.531 ± 0.134	0.905 ± 0.236	0.543 ± 0.069	0.001470308	0.02618866
P05943	Protein S100-A10	S100a10	1.637 ± 0.473	0.698 ± 0.144	0.645 ± 0.186	0.008089812	0.01664138
P06238	Alpha-2-macroglobulin	A2m	1.361 ± 0.047	0.962 ± 0.147	0.666 ± 0.074	0.000792912	0.02149325
P06762	Heme oxygenase 1	Hmox1	1.687 ± 0.622	0.826 ± 0.107	0.491 ± 0.06	0.001963084	0.02485557
P14480	Fibrinogen beta chain	Fgb	1.598 ± 0.364	0.758 ± 0.021	0.66 ± 0.046	0.000529715	0.00160255
P20059	Hemopexin	Hpx	1.409 ± 0.131	0.867 ± 0.12	0.708 ± 0.041	0.000397538	0.00231847
P55926	Acid-sensing ion channel 1	Asic1	1.088 ± 0.062	0.904 ± 0.072	1.007 ± 0.082	0.053200891	0.04568335
Q01984	Histamine *N*-methyltransferase	Hnmt	1.009 ± 0.091	0.822 ± 0.089	1.181 ± 0.001	0.00450368	0.04857169
Q3KR94	Vitronectin	Vtn	1.281 ± 0.201	0.832 ± 0.045	0.885 ± 0.074	0.006881687	0.00815074
Q3T1J1	Eukaryotic translation initiation factor 5A-1	Eif5a	1.288 ± 0.203	0.781 ± 0.069	0.952 ± 0.053	0.004786577	0.00397991
Q499P8	RUS1 family protein C16orf58 homolog	–	1.034 ± 0.009	0.827 ± 0.01	0.951 ± 0.111	0.021371489	0.01825009
Q4FZZ3	Glutathione S-transferase alpha-5	Gsta5	1.898 ± 0.124	0.571 ± 0.047	0.51 ± 0.034	0.0000009	0.0000021
Q4KLL7	Vacuolar protein sorting 4 homolog B	Vps4b	1.182 ± 0.05	0.888 ± 0.037	0.939 ± 0.153	0.031116506	0.03461619
Q4KM86	Gamma-glutamylaminecyclotransferase	Ggact	1.127 ± 0.119	0.905 ± 0.011	0.975 ± 0.048	0.022149449	0.01969118
Q5PPG5	Chga protein	Chga	1.208 ± 0.062	0.948 ± 0.079	0.845 ± 0.061	0.002155917	0.01283758
Q68FY4	Group specific component	Gc	1.316 ± 0.256	0.857 ± 0.014	0.833 ± 0.027	0.004232581	0.00825758
Q6AY91	Nicotinamide riboside kinase 1	Nmrk1	1.119 ± 0.115	0.867 ± 0.047	1.019 ± 0.046	0.013212965	0.01149958
Q7TQ70	Ac1873	Fga	1.558 ± 0.316	0.767 ± 0.056	0.679 ± 0.072	0.000687567	0.00197323

### Subcellular distribution of differentially expressed proteins in the hippocampus

To predict the cellular functions of differentially expressed proteins, the locations of these proteins were analyzed in the current study. The total number of differentially expressed protein in the hippocampus was 117 on day 1 following lithium-pilocarpine administration (compared with controls). Using subcellular location analysis, the distribution was as follows: nucleus (26.5%, 31 proteins), extracellular (25.64%, 30 proteins), cytoplasm (24.79%, 29 proteins), mitochondria (7.69%, 9 proteins), plasma membrane (5.98%, 7 proteins), cytoplasm, nucleus (5.98%, 7 proteins), peroxisome (1.71%, two proteins, heme oxygenase 1, HMOX1; methylsterol monooxygenase 1, MSMO1), endoplasmic reticulum (0.85%, one protein, serine protease inhibitor, SERPINA3N), and cytoskeleton (0.85%, one protein, LisH domain-containing protein ARMC9, ARMC9) (Table 4). On day 3 following lithium-pilocarpine administration (as compared with controls), 59 proteins were distributed in the hippocampus as follows (as determined by subcellular location analysis): extracellular (29%, 17 proteins), the nucleus (22%, 13 proteins), cytoplasm (22%, 13 proteins), mitochondria (10%, six proteins), plasma membrane (8%, 5 proteins), cytoplasm and nucleus (7%, 4 proteins), and endoplasmic reticulum (2%, one protein, cell adhesion molecule L1-like, CHL1) (Table 5). The subcellular location of 51 differentially expressed proteins on day 3 following lithium-pilocarpine administration, compared with day 1, was as follows: the extracellular (25%, 13 proteins), nucleus (25%, 13 proteins), cytoplasm (20%, 10 proteins), plasma membrane (18%, 9 proteins), mitochondria (8%, 4 proteins), peroxisome (2%, 1 protein: HMOX1), and endoplasmic reticulum (2%, 1 protein, SERPINA3N) (Table 6).

**TABLE 4:** Cellular distribution of differentially expression proteins on Day 1 comparing with control (ctrl) by ANOVA analysis in hippocampus.

Subcellular localization	Protein accession	Protein description	Gene name	Mean ± SEM (Day 1)	Mean ± SEM (Day 3)	Mean ± SEM (ctrl)	*P*-value	Post *P*-value (Day 1/ctrl)	Regulated type
Nucleus (26.5%)	F1LX28	Acyl-CoA thioesterase 11	Acot11	0.938 ± 0.062	0.917 ± 0.113	1.16 ± 0.029	0.024426638	0.047601114	Down
	F1M695	YjeF N-terminal domain-containing 3	Yjefn3	0.912 ± 0.041	0.988 ± 0.048	1.108 ± 0.04	0.004614964	0.003820617	Down
	F1M8H7	Actin-associated protein FAM107A	Fam107a	0.768 ± 0.057	1.146 ± 0.023	1.079 ± 0.026	0.0000772	0.000236951	Down
	G3V6S8	Serine/arginine-rich splicing factor 6	Srsf6	1.079 ± 0.038	1.024 ± 0.051	0.898 ± 0.045	0.006340998	0.005933366	Up
	Q9EPX0	Heat shock protein beta-8	Hspb8	1.167 ± 0.061	1.009 ± 0.109	0.826 ± 0.012	0.00311031	0.002534785	Up
	M0R965	Uncharacterized protein	LOC685025	1.265 ± 0.203	0.902 ± 0.081	0.837 ± 0.046	0.007874934	0.008723589	Up
	O35821	Myb-binding protein 1A	Mybbp1a	1.135 ± 0.07	0.994 ± 0.046	0.894 ± 0.047	0.005112057	0.004164782	Up
	P43278	Histone H1.0	H1f0	0.794 ± 0.043	1.039 ± 0.248	1.201 ± 0.12	0.041964103	0.036372871	Down
	P61314	60S ribosomal protein L15	Rpl15	1.124 ± 0.058	1.076 ± 0.181	0.814 ± 0.095	0.039951166	0.044296005	Up
	P62804	Histone H4	Hist1h4b	0.835 ± 0.053	1.053 ± 0.136	1.112 ± 0.108	0.031334673	0.032976035	Down
	Q3B8N7	TSC22 domain family protein 4	Tsc22d4	1.142 ± 0.127	1.005 ± 0.117	0.852 ± 0.059	0.036070482	0.030436262	Up
	Q5U3Y8	Transcription factor BTF3	Btf3	1.108 ± 0.047	0.986 ± 0.043	0.91 ± 0.01	0.001514494	0.001244569	Up
	Q6QI89	Mortality factor 4-like protein 2	Morf4l2	1.175 ± 0.135	1.017 ± 0.016	0.813 ± 0.058	0.003410756	0.002867195	Up
	Q5XI28	Ribonucleoprotein PTB-binding 1	Raver1	1.114 ± 0.088	1.022 ± 0.092	0.87 ± 0.095	0.04198281	0.037441659	Up
	F1LU97	SAM and SH3 domain-containing 1	Sash1	0.852 ± 0.064	0.989 ± 0.095	1.167 ± 0.158	0.031821982	0.026564789	Down
	D4A563	Pseudopodium-enriched atypical kinase 1	Peak1	1.065 ± 0.123	1.035 ± 0.027	0.875 ± 0.041	0.036089869	0.04251408	Up
	D3ZB76	DnaJ (Hsp40) homolog, subfamily B, member 5 (Predicted)	Dnajb5	1.135 ± 0.088	0.994 ± 0.027	0.875 ± 0.012	0.001704428	0.001359745	Up
	D3ZXL5	Nuclear cap-binding subunit 3	Ncbp3	1.124 ± 0.171	1.033 ± 0.107	0.848 ± 0.036	0.048754609	0.045718377	Up
	D4A1U7	Round spermatid basic protein 1	Rsbn1	1.737 ± 0.104	0.621 ± 0.037	0.627 ± 0.048	0.0000018	0.0000031	Up
	A0A0G2K654	Histone cluster 1 H1 family member c	Hist1h1c	0.749 ± 0.085	1.017 ± 0.096	1.254 ± 0.21	0.00792144	0.006647937	Down
	A0A0G2KA11	Phosphatidylinositol-3,4,5-trisphosphate-dependent Rac exchange factor 2	Prex2	0.855 ± 0.071	1.005 ± 0.093	1.111 ± 0.079	0.022575311	0.019371419	Down
	B2GV14	Taxilin alpha	Txlna	1.112 ± 0.038	0.987 ± 0.05	0.903 ± 0.067	0.01015874	0.008415176	Up
	B2RYW7	RCG26543, isoform CRA_b	Srp14	1.118 ± 0.121	0.973 ± 0.098	0.883 ± 0.025	0.046059745	0.039736226	Up
	B5DF45	TNF receptor-associated factor 6	Traf6	0.928 ± 0.056	0.963 ± 0.054	1.119 ± 0.058	0.013804642	0.01466275	Down
	A0A0G2JYD1	Ubiquitin-associated protein 2	Ubap2	1.082 ± 0.051	1.068 ± 0.061	0.854 ± 0.045	0.002524332	0.003693646	Up
	D3ZBN0	Histone H1.5	Hist1h1b	0.86 ± 0.076	0.985 ± 0.056	1.17 ± 0.168	0.035167578	0.029578354	Down
	D3ZGR7	RCG51149	Trir	1.185 ± 0.087	0.886 ± 0.048	0.932 ± 0.092	0.008574615	0.022044093	Up
	D3ZIF0	Zinc finger protein 512	Zfp512	0.688 ± 0.049	1.112 ± 0.331	1.208 ± 0.219	0.046057772	0.048803418	Down
	D3ZML3	Cyclin-dependent kinase 11B	Cdk11b	1.119 ± 0.056	0.979 ± 0.05	0.91 ± 0.028	0.003917502	0.003392973	Up
	D3ZMQ0	MGA, MAX dimerization protein	Mga	1.125 ± 0.108	0.964 ± 0.029	0.919 ± 0.046	0.021165579	0.021150701	Up
	D3ZA21	Pleckstrin homology and RhoGEF domain-containing G3	Plekhg3	0.881 ± 0.129	1.002 ± 0.048	1.128 ± 0.021	0.03315667	0.027745196	Down
Extracellular (25.64%)	P06238	Alpha-2-macroglobulin	A2m	1.361 ± 0.047	0.962 ± 0.147	0.666 ± 0.074	0.000792912	0.000628868	Up
	P14480	Fibrinogen beta chain	Fgb	1.598 ± 0.364	0.758 ± 0.021	0.66 ± 0.046	0.000529715	0.000620191	Up
	P16975	SPARC	Sparc	1.08 ± 0.057	1.053 ± 0.118	0.874 ± 0.033	0.025690482	0.030097449	Up
	P20059	Hemopexin	Hpx	1.409 ± 0.131	0.867 ± 0.12	0.708 ± 0.041	0.000397538	0.000368949	Up
	Q3KR94	Vitronectin	Vtn	1.281 ± 0.201	0.832 ± 0.045	0.885 ± 0.074	0.006881687	0.016683615	Up
	Q5PPG2	Legumain	Lgmn	1.091 ± 0.074	1.091 ± 0.201	0.794 ± 0.054	0.035860633	0.049602943	Up
	Q5XI90	Dynein light chain Tctex-type 3	Dynlt3	0.786 ± 0.171	0.844 ± 0.195	1.387 ± 0.145	0.024011807	0.029094976	Down
	Q63041	Alpha-1-macroglobulin	A1m	1.162 ± 0.186	1.009 ± 0.067	0.86 ± 0.073	0.055049097	0.046839218	Up
	Q68FY4	Group specific component	Gc	1.316 ± 0.256	0.857 ± 0.014	0.833 ± 0.027	0.004232581	0.005987128	Up
	Q6P734	Plasma protease C1 inhibitor	Serping1	1.229 ± 0.027	1 ± 0.169	0.771 ± 0.026	0.003261921	0.002631873	Up
	Q7TQ70	Ac1873	Fga	1.558 ± 0.316	0.767 ± 0.056	0.679 ± 0.072	0.000687567	0.000821385	Up
	Q9QZK5	Serine protease HTRA1	Htra1	1.39 ± 0.485	0.916 ± 0.089	0.715 ± 0.012	0.02251602	0.01927177	Up
	P02803	Metallothionein-1	Mt1	1.531 ± 0.134	0.905 ± 0.236	0.543 ± 0.069	0.001470308	0.001174416	Up
	Q5PPG5	Chga protein	Chga	1.208 ± 0.062	0.948 ± 0.079	0.845 ± 0.061	0.002155917	0.001929208	Up
	P02680	Fibrinogen gamma chain	Fgg	1.561 ± 0.303	0.786 ± 0.03	0.648 ± 0.067	0.000335828	0.00034631	Up
	P35355	Prostaglandin G/H synthase 2	Ptgs2	1.493 ± 0.543	0.985 ± 0.213	0.526 ± 0.071	0.006710757	0.00566147	Up
	G3V8D0	ST8 alpha-*N*-acetyl-neuraminide alpha-2,8-sialyltransferase 3	St8sia3	1.06 ± 0.068	1.089 ± 0.064	0.858 ± 0.071	0.010077793	0.021673603	Up
	A0A0G2JY31	Alpha-1-antiproteinase	Serpina1	1.395 ± 0.324	0.868 ± 0.085	0.735 ± 0.048	0.004597287	0.004442122	Up
	A0A0G2K5J5	Netrin G2	Ntng2	1.165 ± 0.13	0.991 ± 0.084	0.844 ± 0.057	0.013349057	0.010926122	Up
	A0A0G2K624	Brain-derived neurotrophic factor	Bdnf	1.143 ± 0.151	1.08 ± 0.068	0.778 ± 0.089	0.008241653	0.009860647	Up
	A0A0G2K946	SPARC/osteonectin, cwcv and kazal-like domains proteoglycan 2	Spock2	0.924 ± 0.042	0.979 ± 0.013	1.114 ± 0.032	0.001052458	0.000947972	Down
	F1LP80	Neurosecretory protein VGF	Vgf	1.266 ± 0.1	1.041 ± 0.14	0.691 ± 0.042	0.000845572	0.000742592	Up
	F1LR84	Neuronal pentraxin-2	Nptx2	1.134 ± 0.093	1.183 ± 0.374	0.687 ± 0.031	0.031945884	0.049143426	Up
	D3ZHV3	Metallothionein	Mt1m	1.378 ± 0.206	1.092 ± 0.433	0.47 ± 0.032	0.009031572	0.008623115	Up
	F1LST1	Fibronectin	Fn1	1.408 ± 0.031	0.861 ± 0.019	0.721 ± 0.017	0.0000001	0.0000001	Up
	F7EUK4	Kininogen-1	Kng1	1.535 ± 0.102	1.012 ± 0.424	0.44 ± 0.112	0.004142626	0.003524826	Up
	G3V714	Neuroendocrine protein 7B2	Scg5	0.937 ± 0.052	0.982 ± 0.042	1.139 ± 0.074	0.011670151	0.011575582	Down
	G3V7K3	Ceruloplasmin	Cp	1.232 ± 0.189	0.981 ± 0.235	0.799 ± 0.023	0.056300625	0.048127174	Up
	G3V7Z4	Glia-derived nexin	Serpine2	0.812 ± 0.044	1.124 ± 0.077	1.09 ± 0.064	0.001105092	0.002519461	Down
	P01048	T-kininogen 1	Map1	1.385 ± 0.381	1.059 ± 0.589	0.538 ± 0.079	0.043108294	0.037977515	Up
Cytoplasm (24.79%)	A0A0G2JX25	GMP reductase	Gmpr2	0.889 ± 0.106	0.987 ± 0.056	1.118 ± 0.047	0.035514647	0.02978354	Down
	Q9WVJ6	Tissue-type transglutaminase	Tgm2	1.204 ± 0.06	0.982 ± 0.172	0.826 ± 0.015	0.013910287	0.011555675	Up
	Q9EST6	Acidic leucine-rich nuclear phosphoprotein 32 family member B	Anp32b	1.124 ± 0.054	0.959 ± 0.052	0.924 ± 0.042	0.005966698	0.006510222	Up
	Q66HA8	Heat shock protein 105 kDa	Hsph1	1.087 ± 0.031	1.027 ± 0.089	0.885 ± 0.019	0.00900224	0.008450019	Up
	Q5HZA2	Sprouty RTK-signaling antagonist 2	Spry2	1.103 ± 0.117	1.059 ± 0.074	0.845 ± 0.079	0.025569912	0.029512024	Up
	Q4FZZ3	Glutathione S-transferase Alpha-5	Gsta5	1.898 ± 0.124	0.571 ± 0.047	0.51 ± 0.034	0.0000009	0.0000014	Up
	Q3T1J1	Eukaryotic translation initiation factor 5A-1	Eif5a	1.288 ± 0.203	0.781 ± 0.069	0.952 ± 0.053	0.004786577	0.041311914	Up
	Q02765	Cathepsin S	Ctss	1.262 ± 0.097	0.884 ± 0.131	0.855 ± 0.162	0.027558651	0.034687309	Up
	P62912	60S ribosomal protein L32	Rpl32	1.087 ± 0.056	1.034 ± 0.107	0.875 ± 0.047	0.02843089	0.028255172	Up
	P59895	Serine/threonine-protein kinase Nek6	Nek6	1.179 ± 0.166	0.939 ± 0.102	0.887 ± 0.056	0.044020997	0.046634981	Up
	P30713	Glutathione S-transferase theta-2	Gstt2	1.146 ± 0.115	0.982 ± 0.026	0.875 ± 0.067	0.012206229	0.010099762	Up
	P05943	Protein S100-A10	S100a10	1.637 ± 0.473	0.698 ± 0.144	0.645 ± 0.186	0.008089812	0.010515729	Up
	O35760	Isopentenyl-diphosphate Delta-isomerase 1	Idi1	1.104 ± 0.008	1.006 ± 0.074	0.912 ± 0.083	0.037623131	0.031500687	Up
	O35547	Long-chain-fatty-acid–CoA ligase 4	Acsl4	1.108 ± 0.078	0.987 ± 0.047	0.919 ± 0.028	0.013900716	0.011886134	Up
	Q63357	Unconventional myosin-Id	Myo1d	0.909 ± 0.062	1 ± 0.073	1.108 ± 0.087	0.042586877	0.035800118	Down
	F1LZW6	Solute carrier family 25 member 13	Slc25a13	1.176 ± 0.103	0.897 ± 0.052	0.94 ± 0.018	0.003932235	0.011410437	Up
	A0A0G2K7W6	Similar to 60S ribosomal protein L27a	RGD1562402	1.075 ± 0.087	1.045 ± 0.127	0.838 ± 0.059	0.035376846	0.041811288	Up
	A0A0G2QC03	Influenza virus NS1A-binding protein	Ivns1abp	1.123 ± 0.07	1.016 ± 0.076	0.861 ± 0.13	0.043382364	0.038141301	Up
	A0A0H2UHH9	40S ribosomal protein S24	Rps24	1.089 ± 0.039	1.068 ± 0.094	0.837 ± 0.113	0.027333621	0.035307812	Up
	A0A0H2UHP9	RCG39700, isoform CRA_d	Rab6a	1.158 ± 0.065	0.964 ± 0.067	0.881 ± 0.052	0.004523762	0.004012812	Up
	B2RYK2	2-(3-amino-3-carboxypropyl)histidine synthase subunit 1	Dph1	1.127 ± 0.123	0.978 ± 0.057	0.895 ± 0.048	0.03308807	0.028558263	Up
	B1WC40	Nuclear cap-binding protein subunit 2	Ncbp2	1.123 ± 0.015	0.947 ± 0.048	0.934 ± 0.039	0.001977601	0.002786505	Up
	D3ZCB9	Family with sequence similarity 92, member B	Fam92b	0.913 ± 0.052	1.022 ± 0.041	1.112 ± 0.072	0.013409659	0.011172678	Down
	D3ZWS6	*N*(alpha)-acetyltransferase 30, NatC catalytic subunit	Naa30	1.08 ± 0.038	1.057 ± 0.079	0.862 ± 0.029	0.003276391	0.004270506	Up
	D3ZYJ5	GRAM domain-containing 1B	Gramd1b	0.943 ± 0.057	0.933 ± 0.087	1.136 ± 0.04	0.017315829	0.030984495	Down
	D3ZYS7	G3BP stress granule assembly factor 1	G3bp1	1.082 ± 0.037	1.002 ± 0.052	0.896 ± 0.048	0.00805689	0.006715078	Up
	D4A0W1	ER membrane protein complex subunit 4	Emc4	1.13 ± 0.05	0.903 ± 0.115	0.911 ± 0.068	0.02995042	0.049835285	Up
	D3ZBL6	Nucleoporin 160	Nup160	1.123 ± 0.107	1.071 ± 0.135	0.805 ± 0.115	0.034214132	0.039021308	Up
	G3V9W2	Tyrosine-protein kinase	Jak1	1.072 ± 0.047	1.043 ± 0.064	0.885 ± 0.056	0.013013008	0.015147659	Up
Mitochondria (7.69%)	F1LTU4	Ribosome assembly factor mrt4	Mrto4	1.175 ± 0.08	0.911 ± 0.126	0.911 ± 0.048	0.021122368	0.033445134	Up
	Q4KM45	UPF0687 protein C20orf27 homolog	–	1.086 ± 0.077	1.035 ± 0.092	0.864 ± 0.025	0.015008625	0.015455778	Up
	Q4KLZ1	Transmembrane protein 186	Tmem186	0.917 ± 0.053	0.96 ± 0.096	1.133 ± 0.006	0.01949487	0.020474501	Down
	G3V734	2,4-dienoyl CoA reductase 1, mitochondrial, isoform CRA_a	Decr1	1.081 ± 0.053	1.026 ± 0.064	0.896 ± 0.024	0.008005358	0.007554546	Up
	F1LPX0	Mitochondrial intermediate peptidase	Mipep	0.904 ± 0.01	0.992 ± 0.026	1.118 ± 0.038	0.000160503	0.000128185	Down
	D3ZR12	Syntrophin, gamma 2	Sntg2	1.097 ± 0.087	1.068 ± 0.081	0.834 ± 0.093	0.018011349	0.022843965	Up
	B1WBR8	F-box and leucine-rich repeat protein 4	Fbxl4	1.601 ± 0.104	0.684 ± 0.04	0.703 ± 0.049	0.0000058	0.0000113	Up
	A0A0G2K5T6	RNA polymerase I and III subunit C	Polr1c	1.159 ± 0.055	1.003 ± 0.135	0.837 ± 0.029	0.010943722	0.008969521	Up
	A0A0A0MXY7	Uncharacterized protein	–	1.16 ± 0.031	1.038 ± 0.199	0.799 ± 0.042	0.025776779	0.023109735	Up
Plasma membrane (5.98%)	A0A0A0MY39	ATP-binding cassette sub-family B member 9	Abcb9	1.246 ± 0.16	0.922 ± 0.042	0.839 ± 0.071	0.004760401	0.004728188	Up
	A0A0H2UHF5	ATP-sensitive inward rectifier potassium channel 10	Kcnj10	0.766 ± 0.01	0.985 ± 0.237	1.211 ± 0.13	0.027707889	0.023028924	Down
	A0A1W2Q674	Claudin	Cldn10	0.889 ± 0.057	1.014 ± 0.017	1.104 ± 0.084	0.011983829	0.010179599	Down
	D4A017	Transmembrane protein 87A	Tmem87a	1.13 ± 0.101	0.985 ± 0.112	0.883 ± 0.042	0.03823588	0.032409648	Up
	F1M0A0	Anoctamin	Ano3	1.18 ± 0.159	0.997 ± 0.125	0.826 ± 0.115	0.048790639	0.041219969	Up
	P08050	Gap junction alpha-1 protein	Gja1	0.945 ± 0.06	0.898 ± 0.033	1.183 ± 0.08	0.00251446	0.007650803	Down
	Q9Z122	Acyl-CoA 6-desaturase	Fads2	0.791 ± 0.023	1.118 ± 0.129	1.106 ± 0.026	0.001449412	0.002601096	Down
Cytoplasm, nucleus (5.98%)	P04961	Proliferating cell nuclear antigen	Pcna	1.232 ± 0.181	0.965 ± 0.161	0.779 ± 0.034	0.018447444	0.015287816	Up
	Q71UF4	Histone-binding protein RBBP7	Rbbp7	1.109 ± 0.104	0.969 ± 0.038	0.905 ± 0.025	0.016424534	0.014568544	Up
	P52631	Signal transducer and activator of transcription 3	Stat3	1.085 ± 0.07	1.065 ± 0.172	0.815 ± 0.009	0.030420301	0.03815129	Up
	D3ZXL9	Potassium channel tetramerization domain-containing 4	Kctd4	0.931 ± 0.087	0.903 ± 0.032	1.172 ± 0.026	0.002745378	0.006217505	Down
	A0A0G2K2E2	Antizyme inhibitor 2	Azin2	1.092 ± 0.154	1.111 ± 0.018	0.804 ± 0.125	0.026624522	0.046602584	Up
	A0A096MJE3	G1 to S phase transition 2	Gspt2	1.13 ± 0.073	0.946 ± 0.015	0.93 ± 0.104	0.037113703	0.044872317	Up
	B2RYI0	WD repeat-containing protein 91	Wdr91	0.899 ± 0.045	1.027 ± 0.023	1.084 ± 0.012	0.001055191	0.000976489	Down
Peroxisome (1.71%)	P06762	Heme oxygenase 1	Hmox1	1.687 ± 0.622	0.826 ± 0.107	0.491 ± 0.06	0.001963084	0.001591598	Up
	O35532	Methylsterol monooxygenase 1	Msmo1	1.117 ± 0.046	0.982 ± 0.083	0.906 ± 0.036	0.012941343	0.011098348	Up
Cytoskeleton (0.85%)	A0A0G2K9J8	LisH domain-containing protein ARMC9	Armc9	1.108 ± 0.163	1.033 ± 0.035	0.853 ± 0.061	0.041736227	0.04131062	Up
Endoplasmic reticulum (0.85%)	A0A0H2UHI5	Serine protease inhibitor	Serpina3n	1.592 ± 0.268	0.795 ± 0.223	0.607 ± 0.066	0.002321611	0.002250448	Up

**TABLE 5:** Cellular distribution of differentially expression proteins on Day 3 comparing with control (ctrl) by ANOVA analysis in hippocampus.

Subcellular localization	Protein accession	Protein description	Gene name	Mean ± SEM (Day 1)	Mean ± SEM (Day 3)	Mean ± SEM (ctrl)	*P*-value	Post *P*-value (Day 3/ctrl)	Regulated type
Extracellular (29%)	O35263	Platelet-activating factor acetylhydrolase IB subunit gamma	Pafah1b3	1.003 ± 0.072	1.135 ± 0.141	0.886 ± 0.049	0.038926	0.032614	Up
	Q6P734	Plasma protease C1 inhibitor	Serping1	1.229 ± 0.027	1 ± 0.169	0.771 ± 0.026	0.003262	0.044507	Up
	Q5XI90	Dynein light chain Tctex-type 3	Dynlt3	0.786 ± 0.171	0.844 ± 0.195	1.387 ± 0.145	0.024012	0.04766	Down
	Q3V5 × 8	Endonuclease G	Endog	0.962 ± 0.022	0.921 ± 0.093	1.128 ± 0.077	0.028168	0.028427	Down
	P35355	Prostaglandin G/H synthase 2	Ptgs2	1.493 ± 0.543	0.985 ± 0.213	0.526 ± 0.071	0.006711	0.048988	Up
	P06238	Alpha-2-macroglobulin	A2m	1.361 ± 0.047	0.962 ± 0.147	0.666 ± 0.074	0.000793	0.018911	Up
	O35314	Secretogranin-1	Chgb	1.022 ± 0.02	1.093 ± 0.116	0.88 ± 0.043	0.021703	0.020195	Up
	G3V8D0	ST8 alpha-N-acetyl-neuraminide alpha-2,8-sialyltransferase 3	St8sia3	1.06 ± 0.068	1.089 ± 0.064	0.858 ± 0.071	0.010078	0.012504	Up
	P02803	Metallothionein-1	Mt1	1.531 ± 0.134	0.905 ± 0.236	0.543 ± 0.069	0.00147	0.041145	Up
	F1LR84	Neuronal pentraxin-2	Nptx2	1.134 ± 0.093	1.183 ± 0.374	0.687 ± 0.031	0.031946	0.045781	Up
	F1LP80	Neurosecretory protein VGF	Vgf	1.266 ± 0.1	1.041 ± 0.14	0.691 ± 0.042	0.000846	0.006023	Up
	F7EUK4	Kininogen-1	Kng1	1.535 ± 0.102	1.012 ± 0.424	0.44 ± 0.112	0.004143	0.030278	Up
	D3ZHV3	Metallothionein	Mt1m	1.378 ± 0.206	1.092 ± 0.433	0.47 ± 0.032	0.009032	0.034986	Up
	D3ZDM7	D-aspartate oxidase	Ddo	0.99 ± 0.075	1.108 ± 0.069	0.908 ± 0.08	0.048742	0.041551	Up
	A0A0G2QC22	PAXX, non-homologous end joining factor	Paxx	0.973 ± 0.074	0.904 ± 0.043	1.14 ± 0.097	0.020131	0.018321	Down
	A0A0G2K624	Brain-derived neurotrophic factor	Bdnf	1.143 ± 0.151	1.08 ± 0.068	0.778 ± 0.089	0.008242	0.019242	Up
	A0A0G2K3 × 6	Uncharacterized protein	–	1.014 ± 0.071	0.857 ± 0.118	1.141 ± 0.097	0.03683	0.031574	Down
Cytoplasm (22%)	A0A0G2JWD6	AP-3 complex subunit beta	Ap3b1	1.045 ± 0.022	1.076 ± 0.097	0.881 ± 0.082	0.037566	0.043753	Up
	P55063	Heat shock 70 kDa protein 1-like	Hspa1l	0.807 ± 0.104	1.472 ± 0.486	0.731 ± 0.082	0.02445	0.028127	Up
	P18484	AP-2 complex subunit alpha-2	Ap2a2	0.976 ± 0.035	0.888 ± 0.043	1.072 ± 0.069	0.011174	0.009119	Down
	G3V8F9	Alpha-methylacyl-CoA racemase	Amacr	1.096 ± 0.053	0.867 ± 0.054	1.048 ± 0.091	0.012544	0.034582	Down
	F1LV07	Dynein, axonemal, heavy chain 9	Dnah9	0.973 ± 0.045	0.916 ± 0.067	1.122 ± 0.086	0.025205	0.023287	Down
	F1LS29	Calpain-1 catalytic subunit	Capn1	0.999 ± 0.083	1.103 ± 0.06	0.902 ± 0.064	0.035158	0.029378	Up
	Q01984	Histamine *N*-methyltransferase	Hnmt	1.009 ± 0.091	0.822 ± 0.089	1.181 ± 0.001	0.004504	0.003676	Down
	D4A8F2	Ras suppressor protein 1	Rsu1	0.99 ± 0.053	1.117 ± 0.107	0.9 ± 0.082	0.051994	0.04422	Up
	D3ZYJ5	GRAM domain-containing 1B	Gramd1b	0.943 ± 0.057	0.933 ± 0.087	1.136 ± 0.04	0.017316	0.023376	Down
	D3ZWS6	N(alpha)-acetyltransferase 30, NatC catalytic subunit	Naa30	1.08 ± 0.038	1.057 ± 0.079	0.862 ± 0.029	0.003276	0.007307	Up
	A0A0H2UHH9	40S ribosomal protein S24	Rps24	1.089 ± 0.039	1.068 ± 0.094	0.837 ± 0.113	0.027334	0.048618	Up
	A0A0G2K890	Ezrin	Ezr	0.988 ± 0.066	1.147 ± 0.197	0.853 ± 0.029	0.049278	0.041561	Up
	F1LQ22	Unconventional SNARE in the ER 1	Use1	1.046 ± 0.054	1.082 ± 0.072	0.871 ± 0.109	0.040227	0.045588	Up
Nucleus (22%)	P60825	Cold-inducible RNA-binding protein	Cirbp	1.006 ± 0.015	0.916 ± 0.054	1.113 ± 0.088	0.017996	0.014819	Down
	Q6QI89	Mortality factor 4-like protein 2	Morf4l2	1.175 ± 0.135	1.017 ± 0.016	0.813 ± 0.058	0.003411	0.027886	Up
	Q9EPX0	Heat shock protein beta-8	Hspb8	1.167 ± 0.061	1.009 ± 0.109	0.826 ± 0.012	0.00311	0.035314	Up
	Q5XI44	X-ray repair complementing defective repair in Chinese hamster cells 4	Xrcc4	1.026 ± 0.027	1.085 ± 0.047	0.893 ± 0.049	0.004051	0.003805	Up
	Q4W1H3	Myosin 9b	Myo9b	0.982 ± 0.14	1.124 ± 0.046	0.895 ± 0.048	0.052509	0.045919	Up
	P11530	Dystrophin	Dmd	1.006 ± 0.089	0.875 ± 0.075	1.098 ± 0.043	0.025788	0.022128	Down
	A0A0G2JZ56	Ankyrin 2	Ank2	0.966 ± 0.055	0.922 ± 0.048	1.126 ± 0.055	0.00853	0.008607	Down
	F1LX28	Acyl-CoA thioesterase 11	Acot11	0.938 ± 0.062	0.917 ± 0.113	1.16 ± 0.029	0.024427	0.029911	Down
	D3ZB30	Polypyrimidine tract binding protein 1, isoform CRA_c	Ptbp1	1.029 ± 0.117	1.087 ± 0.049	0.893 ± 0.019	0.038545	0.035923	Up
	A0A0G2K2R0	Uncharacterized protein	–	0.991 ± 0.047	0.897 ± 0.053	1.123 ± 0.046	0.00408	0.003317	Down
	A0A0G2K1W1	RAB11 family-interacting protein 5	Rab11fip5	1.054 ± 0.051	1.072 ± 0.066	0.885 ± 0.091	0.036543	0.04601	Up
	A0A0G2JYD1	Ubiquitin-associated protein 2	Ubap2	1.082 ± 0.051	1.068 ± 0.061	0.854 ± 0.045	0.002524	0.004909	Up
	G3V7R4	Forkhead box protein O1	Foxo1	0.969 ± 0.086	0.906 ± 0.07	1.136 ± 0.101	0.044595	0.041773	Down
Mitochondria (10%)	Q499N5	Acyl-CoA synthetase family member 2, mitochondrial	Acsf2	1.011 ± 0.034	0.888 ± 0.089	1.1 ± 0.043	0.017128	0.014612	Down
	D4A7T8	Family with sequence similarity 81, member A	Fam81a	1.005 ± 0.04	0.885 ± 0.076	1.104 ± 0.11	0.041193	0.035271	Down
	D3ZZN3	Acetyl-coenzyme A synthetase	Acss1	1.014 ± 0.101	0.899 ± 0.068	1.094 ± 0.049	0.05326	0.046168	Down
	A0A0G2K6H2	Maleylacetoacetate isomerase	Gstz1	1.013 ± 0.125	0.808 ± 0.107	1.146 ± 0.12	0.031248	0.027634	Down
	A0A0G2K1N9	Selenoprotein O	Selenoo	1.048 ± 0.065	0.868 ± 0.077	1.093 ± 0.077	0.018983	0.02072	Down
	D3ZR12	Syntrophin, gamma 2	Sntg2	1.097 ± 0.087	1.068 ± 0.081	0.834 ± 0.093	0.018011	0.034845	Up
Plasma membrane (8%)	Q925D4	Transmembrane protein 176B	Tmem176b	1.05 ± 0.205	1.242 ± 0.217	0.714 ± 0.188	0.045113	0.041998	Up
	D3ZBN4	Ergosterol biosynthesis 28 homolog	Erg28	1.058 ± 0.123	0.863 ± 0.068	1.094 ± 0.078	0.037958	0.042839	Down
	P08050	Gap junction alpha-1 protein	Gja1	0.945 ± 0.06	0.898 ± 0.033	1.183 ± 0.08	0.002514	0.002788	Down
	P29534	Vascular cell adhesion protein 1	Vcam1	1.013 ± 0.036	0.891 ± 0.088	1.071 ± 0.019	0.022541	0.020949	Down
	Q9EP88	Brain mitochondrial carrier protein BMCP1	Slc25a14	0.986 ± 0.085	0.898 ± 0.055	1.125 ± 0.117	0.051371	0.0442	Down
Cytoplasm, nucleus (7%)	D3ZXL9	Potassium channel tetramerization domain-containing 4	Kctd4	0.931 ± 0.087	0.903 ± 0.032	1.172 ± 0.026	0.002745	0.003575	Down
	F1LVR8	Myocardin-related transcription factor A	Mrtfa	1.045 ± 0.114	1.076 ± 0.033	0.885 ± 0.037	0.032501	0.035827	Up
	A0A0G2K2E2	Antizyme inhibitor 2	Azin2	1.092 ± 0.154	1.111 ± 0.018	0.804 ± 0.125	0.026625	0.034925	Up
	B1WBV1	Axin interactor, dorsalization-associated	Aida	0.995 ± 0.067	1.117 ± 0.09	0.891 ± 0.064	0.027139	0.022541	Up
Endoplasmic reticulum (2%)	M0RC17	Cell adhesion molecule L1-like	Chl1	0.98 ± 0.036	1.115 ± 0.112	0.914 ± 0.066	0.045955	0.040653	Up

**TABLE 6:** Cellular distribution of differentially expression proteins on Day 3 comparing with Day 1 by ANOVA analysis in hippocampus.

Subcellular localization	Protein accession	Protein description	Gene name	Mean ± SEM (Day 1)	Mean ± SEM (Day 3)	Mean ± SEM (ctrl)	*P*-value	Post *P*-value (Day 3/Day 1)	Regulated type
Extracellular (25%)	Q3KR94	Vitronectin	Vtn	1.281 ± 0.201	0.832 ± 0.045	0.885 ± 0.074	0.006881687	0.008150737	Down
	Q7TQ70	Ac1873	Fga	1.558 ± 0.316	0.767 ± 0.056	0.679 ± 0.072	0.000687567	0.001973234	Down
	Q68FY4	Group specific component	Gc	1.316 ± 0.256	0.857 ± 0.014	0.833 ± 0.027	0.004232581	0.008257581	Down
	Q5PPG5	Chga protein	Chga	1.208 ± 0.062	0.948 ± 0.079	0.845 ± 0.061	0.002155917	0.012837584	Down
	Q4KM86	Gamma-glutamylaminecyclotransferase	Ggact	1.127 ± 0.119	0.905 ± 0.011	0.975 ± 0.048	0.022149449	0.019691184	Down
	P20059	Hemopexin	Hpx	1.409 ± 0.131	0.867 ± 0.12	0.708 ± 0.041	0.000397538	0.002318468	Down
	F1LST1	Fibronectin	Fn1	1.408 ± 0.031	0.861 ± 0.019	0.721 ± 0.017	0.0000001	0.0000007	Down
	P06238	Alpha-2-macroglobulin	A2m	1.361 ± 0.047	0.962 ± 0.147	0.666 ± 0.074	0.000792912	0.021493248	Down
	P02803	Metallothionein-1	Mt1	1.531 ± 0.134	0.905 ± 0.236	0.543 ± 0.069	0.001470308	0.026188657	Down
	P02680	Fibrinogen gamma chain	Fgg	1.561 ± 0.303	0.786 ± 0.03	0.648 ± 0.067	0.000335828	0.001396984	Down
	G3V7Z4	Glia-derived nexin	Serpine2	0.812 ± 0.044	1.124 ± 0.077	1.09 ± 0.064	0.001105092	0.001493145	Up
	A0A0G2JY31	Alpha-1-antiproteinase	Serpina1	1.395 ± 0.324	0.868 ± 0.085	0.735 ± 0.048	0.004597287	0.018747055	Down
	P14480	Fibrinogen beta chain	Fgb	1.598 ± 0.364	0.758 ± 0.021	0.66 ± 0.046	0.000529715	0.001602549	Down
Nucleus (25%)	Q9R1K8	RAS guanyl-releasing protein 1	Rasgrp1	0.861 ± 0.097	1.145 ± 0.048	1.003 ± 0.111	0.02849426	0.023748075	Up
	Q4KLL7	Vacuolar protein sorting 4 homolog B	Vps4b	1.182 ± 0.05	0.888 ± 0.037	0.939 ± 0.153	0.031116506	0.034616193	Down
	M0R965	Uncharacterized protein	LOC685025	1.265 ± 0.203	0.902 ± 0.081	0.837 ± 0.046	0.007874934	0.021313333	Down
	H9N1L3	BCL11B, BAF complex component	Bcl11b	0.9 ± 0.066	1.101 ± 0.058	1.005 ± 0.093	0.045468626	0.0383876	Up
	G3V917	Protein TANC1	Tanc1	0.922 ± 0.044	1.167 ± 0.16	0.941 ± 0.008	0.033418706	0.041789671	Up
	F1M8H7	Actin-associated protein FAM107A	Fam107a	0.768 ± 0.057	1.146 ± 0.023	1.079 ± 0.026	0.0000772	0.0000932	Up
	P97577	Fasciculation and elongation protein zeta-1	Fez1	0.877 ± 0.045	1.057 ± 0.073	1.015 ± 0.062	0.021340053	0.022070258	Up
	D4A1U7	Round spermatid basic protein 1	Rsbn1	1.737 ± 0.104	0.621 ± 0.037	0.627 ± 0.048	0.0000018	0.0000030	Down
	D3ZQN3	PNMA family member 8B	Pnma8b	0.887 ± 0.059	1.086 ± 0.084	1.034 ± 0.05	0.021802957	0.021886231	Up
	D3ZGR7	RCG51149	Trir	1.185 ± 0.087	0.886 ± 0.048	0.932 ± 0.092	0.008574615	0.009698347	Down
	A0A0G2JVP4	Immunoglobulin heavy constant mu	Ighm	0.859 ± 0.071	1.182 ± 0.075	0.969 ± 0.102	0.011212146	0.009630313	Up
	A0A0G2JTL7	RBR-type E3 ubiquitin transferase	Ankib1	1.126 ± 0.104	0.893 ± 0.098	0.984 ± 0.067	0.054241554	0.046503851	Down
	D4A542	G protein-coupled receptor-associated sorting protein 2	Gprasp2	0.893 ± 0.083	1.087 ± 0.052	1.03 ± 0.043	0.024300816	0.023329603	Up
Cytoplasm (20%)	A0A0H2UHP9	RCG39700, isoform CRA_d	Rab6a	1.158 ± 0.065	0.964 ± 0.067	0.881 ± 0.052	0.004523762	0.025765079	Down
	Q6AY91	Nicotinamide riboside kinase 1	Nmrk1	1.119 ± 0.115	0.867 ± 0.047	1.019 ± 0.046	0.013212965	0.011499578	Down
	Q3T1J1	Eukaryotic translation initiation factor 5A-1	Eif5a	1.288 ± 0.203	0.781 ± 0.069	0.952 ± 0.053	0.004786577	0.003979909	Down
	Q01984	Histamine *N*-methyltransferase	Hnmt	1.009 ± 0.091	0.822 ± 0.089	1.181 ± 0.001	0.00450368	0.048571688	Down
	P05943	Protein S100-A10	S100a10	1.637 ± 0.473	0.698 ± 0.144	0.645 ± 0.186	0.008089812	0.016641383	Down
	Q4FZZ3	Glutathione S-transferase alpha-5	Gsta5	1.898 ± 0.124	0.571 ± 0.047	0.51 ± 0.034	0.000001	0.0000021	Down
	F1LZW6	Solute carrier family 25 member 13	Slc25a13	1.176 ± 0.103	0.897 ± 0.052	0.94 ± 0.018	0.003932235	0.004370967	Down
	D4A0W1	ER membrane protein complex subunit 4	Emc4	1.13 ± 0.05	0.903 ± 0.115	0.911 ± 0.068	0.02995042	0.04036656	Down
	D3ZA45	Autophagy-related protein 13	Atg13	0.91 ± 0.04	1.121 ± 0.071	0.975 ± 0.057	0.010603183	0.009463803	Up
	G3V8F9	Alpha-methylacyl-CoA racemase	Amacr	1.096 ± 0.053	0.867 ± 0.054	1.048 ± 0.091	0.012544079	0.013376436	Down
Plasma membrane (18%)	Q499P8	RUS1 family protein C16orf58 homolog	–	1.034 ± 0.009	0.827 ± 0.01	0.951 ± 0.111	0.021371489	0.018250087	Down
	A0A0A0MY39	ATP-binding cassette sub-family B member 9	Abcb9	1.246 ± 0.16	0.922 ± 0.042	0.839 ± 0.071	0.004760401	0.017790941	Down
	A0A0U1RRP1	Synaptogyrin-1 (Fragment)	Syngr1	0.895 ± 0.062	1.085 ± 0.076	1.031 ± 0.063	0.030283802	0.029341536	Up
	D3Z899	Mitoguardin 2	Miga2	1.184 ± 0.136	0.82 ± 0.108	1.007 ± 0.085	0.017770309	0.014819532	Down
	D4AAI8	Adhesion G protein-coupled receptor G7	Adgrg7	0.916 ± 0.046	1.108 ± 0.053	0.982 ± 0.059	0.012669651	0.011005002	Up
	D4ABX8	Leucine-rich repeat and fibronectin type-III domain-containing protein 4	Lrfn4	1.16 ± 0.076	0.832 ± 0.097	1.022 ± 0.15	0.035324956	0.03040222	Down
	F1LTD7	DENN domain-containing 4C	Dennd4c	1.096 ± 0.047	0.896 ± 0.105	1.014 ± 0.057	0.055019514	0.048244363	Down
	P55926	Acid-sensing ion channel 1	Asic1	1.088 ± 0.062	0.904 ± 0.072	1.007 ± 0.082	0.053200891	0.04568335	Down
	Q9Z122	Acyl-CoA 6-desaturase	Fads2	0.791 ± 0.023	1.118 ± 0.129	1.106 ± 0.026	0.001449412	0.002342176	Up
Mitochondria (8%)	F1LTU4	Ribosome assembly factor mrt4	Mrto4	1.175 ± 0.08	0.911 ± 0.126	0.911 ± 0.048	0.021122368	0.031016637	Down
	D4A469	Sestrin 3	Sesn3	0.872 ± 0.137	1.219 ± 0.119	0.919 ± 0.078	0.024385703	0.026855355	Up
	B1WBR8	F-box and leucine-rich repeat protein 4	Fbxl4	1.601 ± 0.104	0.684 ± 0.04	0.703 ± 0.049	0.0000058	0.0000093	Down
	A0A0G2K1N9	Selenoprotein O	Selenoo	1.048 ± 0.065	0.868 ± 0.077	1.093 ± 0.077	0.018982576	0.046834353	Down
Endoplasmic reticulum (2%)	A0A0H2UHI5	Serine protease inhibitor	Serpina3n	1.592 ± 0.268	0.795 ± 0.223	0.607 ± 0.066	0.002321611	0.010093209	Down
Peroxisome (2%)	P06762	Heme oxygenase 1	Hmox1	1.687 ± 0.622	0.826 ± 0.107	0.491 ± 0.06	0.001963084	0.024855569	Down

These results show that the number of subcellularly distributed proteins decreased by more than half on day 3 compared with that found on day 1 following lithium-pilocarpine administration, indicating that different cellular functions are required during seizure progression. Indeed, SERPINA3N was the only protein to be regulated in the endoplasmic reticulum, ARMC9 was the only proteins to be modulated in the cytoskeleton on day 1 following lithium-pilocarpine administration compared with controls (Table 4), and CHL1 was the only proteins to be modulated in the endoplasmic reticulum on day 3 following lithium-pilocarpine administration compared with controls (Table 5).

All of these results suggest that cellular function in the hippocampus following seizures is possibly regulated in a differential manner. On day 3 following lithium-pilocarpine administration (compared with day 1), shared 21 proteins among differentially regulated proteins distributed in subcellular locations, representing a small portion of regulated proteins (Tables 4, 5). On day 3 following lithium-pilocarpine administration (compared with day 1), 22 proteins were the same within day 1 following lithium-pilocarpine administration compared with controls (in evaluations conducted *via* subcellular analysis, Tables 4, 6), and only four proteins were same within day 3 following lithium-pilocarpine administration compared with controls (Tables 5, 6), suggesting that cells are recruited on a large scale in the mediation of early versus late seizure activity in the hippocampus. Moreover, alpha-2-macroglobulin (A2M), and Metallothionein-1 (MT1) were observed to be regulated on both day 1 and day 3 following lithium-pilocarpine administration (as compared with controls). Specifically, A2M, and MT1 were upregulated on both days, but the increases on day 3 were lower than that on day 1. To better understand the functionality of differentially expressed proteins, GO and KEGG pathway-based enrichment analyses were performed, as described below.

### Gene ontology annotation and analysis of differentially expressed proteins

**FIGURE 2:**
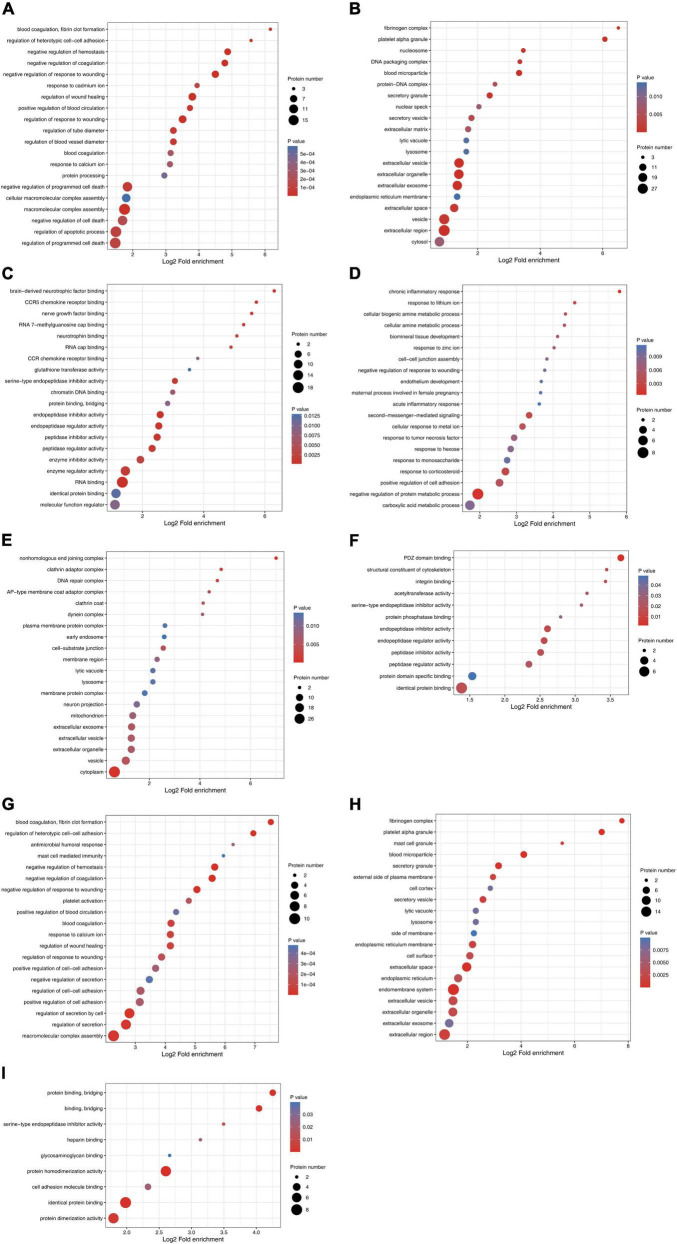
Functional enrichment and cluster analysis of differentially expressed proteins in hippocampus in lithium-pilocarpine induced acute epileptic rat. **(A)** GO “biological process” enrichment of differentially expressed proteins on Day 1 comparing with control (ctrl) in hippocampus. **(B)** GO “cellular component” enrichment of differentially expressed proteins on Day 1 comparing with control (ctrl) in hippocampus. **(C)** GO “molecular function” enrichment of differentially expressed proteins on Day 1 comparing with control (ctrl) in hippocampus. **(D)** GO “biological process” enrichment of differentially expressed proteins on Day 3 comparing with control (ctrl) in hippocampus. **(E)** GO “cellular component” enrichment of differentially expressed proteins on Day 3 comparing with control (ctrl) in hippocampus. **(F)** GO “molecular function” enrichment of differentially expressed proteins on Day 3 comparing with control (ctrl) in hippocampus. **(G)** GO “biological process” enrichment of differentially expressed proteins on Day 3 comparing with Day 1 in hippocampus. **(H)** GO “cellular component” enrichment of differentially expressed proteins on Day 3 comparing with Day 1 in hippocampus. **(I)** GO “molecular function” enrichment of differentially expressed proteins on Day 3 comparing with Day 1 in hippocampus.

The number of differentially expressed proteins was calculated using level 2 GO terms according to GO annotation information, which contributed to characterization of their bio-functions. On day 1 following lithium-pilocarpine administration (compared with controls), 18 proteins were mapped within macromolecular complex assembly, 17 proteins were mapped within regulation of programmed cell death or regulation of apoptotic process, 20 proteins were mapped within negative regulation of programmed cell death, and five proteins were mapped within positive regulation of blood circulation in the “Biological Process” category (Figure 2A). A total of 18 proteins were clustered within RNA binding, six proteins were classified as peptidase inhibitor activity, endopeptidase regulator activity or endopeptidase inhibitor activity in the “Molecular Function” category (Figure 2B). A total of 101 proteins participated in extracellular exosome, vesicle, organelle, and region in the “Cellular Component” category (Figure 2C).

All these results indicate that, on day 1 following lithium-pilocarpine administration, macromolecular complex assembly, cell death and apoptotic process, blood circulation, RNA binding, and the extracellular regulation, were the main regulation targets in the hippocampus. On day 3 following lithium-pilocarpine administration (compared with controls), eight proteins were mapped within the negative regulation of protein metabolic process, four proteins were clustered within positive regulation of cell adhesion, or response to corticosteroid in the “Biological Process” category (Figure 2D). Thirty-three proteins were mapped to cytoplasm, 14 proteins were predicted within vesicle, and 4 proteins were mapped within cell-substrate junction in the “Cellular Component” category (Figure 2E). Moreover, seven proteins were mapped to identical protein binding, and three proteins were clustered within PDZ domain binding, endopeptidase inhibitor activity, endopeptidase regulator activity, or peptidase inhibitor activity in the “Molecular Function” category (Figure 2F).

In addition, all these results suggest that, on day 3 following lithium-pilocarpine administration, protein metabolic process rather than macromolecular complex assembly and cell death were affected in the hippocampus. In addition, on day 3 following the induced seizures (compared with day 1 following seizures), 10 proteins were clustered in macromolecular complex assembly, 8 proteins were mapped in the regulation of secretion by the cell, and 5 proteins were classified in the regulation of cell–cell adhesion in the “Biological Process” category (Figure 2G). Moreover, 17 proteins were mapped to the endomembrane system, 16 proteins were mapped to the extracellular region, 11 proteins were clustered within extracellular space, and 5 proteins were mapped to the secretory granule, or blood microparticle in the “Cellular Component” category (Figure 2H). Eight proteins were predicted in identical protein binding, seven proteins were mapped to protein homodimerization or dimerization activity, and three proteins were predicted in cell adhesion molecule binding or protein binding and bridging in the “Molecular Function” category (Figure 2I).

These results show that, on day 3 following lithium-pilocarpine administration (compared with day 1), the ECM, the constitution of plasma membranes, cell contact and secretion, and protein complexes in the hippocampus were altered in the development of seizure events.

### Distribution and KEGG function analysis of differentially expressed proteins

KEGG pathway enrichment cluster analysis was performed to assess the possible involvement of signaling pathways in seizure events. On day 1 following lithium-pilocarpine administration, as compared with controls, 10 proteins were found to be clustered in the signaling pathway in cancer (regulating sustained angiogenesis and evading apoptosis); nine proteins were upregulated, indicating the cell death processes for further seizure events. This is in line with the findings of the GO analysis presented above.

**FIGURE 3:**
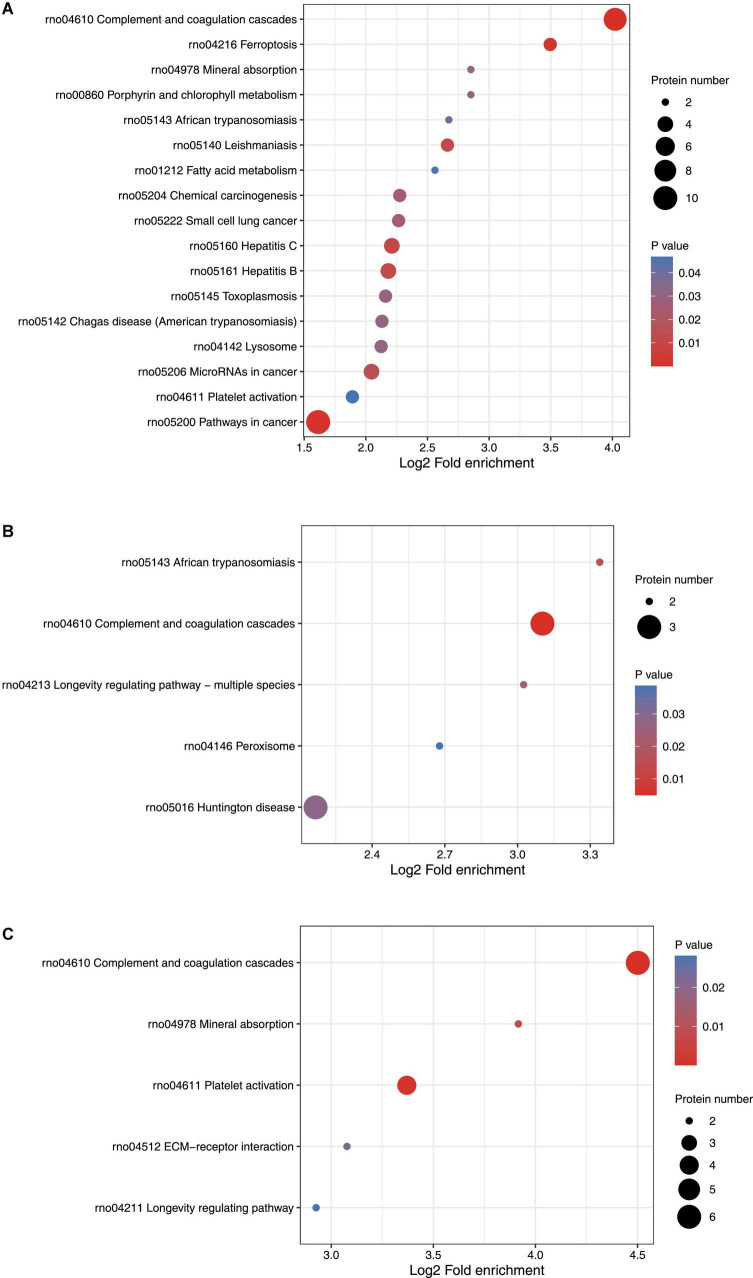
KEGG function analysis of differentially expressed proteins. **(A)** KEGG function analysis of differentially expressed proteins on Day 1 comparing with control (ctrl) in hippocampus. **(B)** KEGG function analysis of differentially expressed proteins on Day 3 comparing with control (ctrl) in hippocampus. **(C)** KEGG function analysis of differentially expressed proteins on Day 3 comparing with Day 1 in hippocampus.

Moreover, nine upregulated proteins were predicted in complement and coagulation cascades, which participate in inflammation response, cell lysis, and phagocytosis, and four proteins were mapped to pathways relevant to MicroRNAs in cancer. Three proteins (Fibronectin, FN; Kininogen 1, KNG1; T-kininogen 1, MAP1), all increased, represent pivotal pathways for modulating seizures; namely, regulatory processes for filopodia and lamellipodia of the actin cytoskeleton, the PI3K-Akt signaling pathway, platelet activation, cell adhesion molecules (CAMs) pathway, the nucleotide-binding oligomerization domain (NOD)-like receptor signaling pathway, the sphingolipid signaling pathway, the hypoxia-inducible factor 1 (HIF-1) signaling pathway, lysosomes, ECM-receptor interaction, and inflammatory mediator regulation of transient receptor potential (TRP) channels were all regulated (Figure 3A).

On day 3 following lithium-pilocarpine administration (compared with controls), the only indicated pathways were those relevant to Huntington’s disease, TNF signaling, NF-kappa B signaling, complement and coagulation cascades, MAPK signaling, PI3K-Akt signaling, apoptosis, regulation of the actin cytoskeleton, and protein processing in the endoplasmic reticulum. However, there were no more than four proteins in each pathway, indicating that the involved pathways were possibly less active on day 3 than on day 1 following lithium-pilocarpine administration (as compared with controls) (Figure 3B).

On day 3 following lithium-pilocarpine administration (as compared with on day 1), six proteins were found to participate in complement and coagulation cascades, four proteins were mapped in the pathway of cancer, or platelet activation. The minority of proteins were predicted to regulate were the PI3K-Akt signaling pathway, mineral absorption, proteoglycans (mediators of cancer tissue mechanics), and ECM-receptor interaction (in which only two proteins were involved) (Figure 3C).

## Discussion

Epilepsy in an umbrella term that describes varieties of convulsive disorders. Studies on epileptogenesis have been conducted in animal models of SE, such as lithium-pilocarpine induced rodent animal models. The hippocampus is the primary site of epileptic activity. Therefore, we conducted a study to detect global protein expression in the hippocampus in SE induced by lithium-pilocarpine in order to understand seizure events in regard to late phase epileptogenesis. In our study, we identified 6,157 proteins in total and quantified 5,593 proteins. Most of the differentially expressed proteins were predicted to be upregulated in the hippocampus on days 1 and 3 following lithium-pilocarpine administration, indicating that protein expression was likely to be enhanced rather than prohibited in the modulation of seizures within SE. Moreover, the number of enhanced proteins in the hippocampus decreased by more than half from day 1 to day 3, and only a small portion of proteins were the same when comparing these timepoints, suggesting that different molecules and pathways are involved in epilepsy events occurring from day 1 to day 3 following lithium-pilocarpine administration.

In our study, several differential expression proteins involved in the phasing of seizure events, such as EIF5A. Previous work has demonstrated that reduced hypusinated EIF5A causes neurological impairment, including seizures (Ganapathi et al., 2019). Further, a prior study demonstrated that EIF5A regulated neuronal survival and growth (Huang et al., 2007), indicating that EIF5A and other molecules are upregulated in protecting neurons against the damage caused by seizures. Several roles of EIF5A have been reported; for example, neuronal apoptosis is regulated by EIF5A/p53 (Li et al., 2004), axonal growth of dorsal root ganglion (DRG) neurons is stimulated by brain-derived neurotrophic factor (BDNF)/arginase I/EIF5A/cAMP (Cai et al., 2002; Huang et al., 2007), and EIF5A variants have been found to cause several disorders, such as developmental delay, intellectual disability, facial dysmorphisms, and microcephaly (Park et al., 2022). Of note, EIF5A stabilizes ribosome components and promotes mRNA translation termination and elongation (Chen et al., 1996; Schuller et al., 2017). In our study, the levels of several ribosome components increased on day 1 following lithium-pilocarpine administration, suggesting the potential function of EIF5A in stabilizing ribosomes for mRNA transcription. However, on day 3 following lithium-pilocarpine administration, the levels of EIF5A and ribosome components were decreased and were similar to those of controls, suggesting neuron loss, apoptosis, and degeneration in the hippocampus.

In intractable epilepsy (IE), the neural network is reorganized to properly transduce signals; this is a prominent pathological change that leads to the transient expression of several molecules, such as netrin G2, fibronectin (Fn), and vitronectin. Netrin G2 has been shown to modulate synapse formation and neurite outgrowth (Lin et al., 2003; Kim et al., 2006). Moreover, the overexpression of netrin G2 within excitatory neurons in patients with IE and in the hippocampus of lithium-pilocarpine induced rat models is assumed to be commensurate with abnormal synapse development and neuron migration (Woo et al., 2009; Pan et al., 2010); this is in line with our findings reported here, as epileptic discharges and spreading are supported by abnormal synapses (Buckmaster et al., 2002). Moreover, synaptic reorganization promotes the development of the excitatory loop, and Fn and its integrin receptor are known to participate in the pathophysiology of epilepsy (Gall and Lynch, 2004; Dityatev and Fellin, 2008; Wu and Reddy, 2012; Pitkanen et al., 2014). Other studies in addition to our own have shown that Fn expression is increased in the hippocampus after a first behavioral seizure (Hoffman et al., 1998; Wu et al., 2017). Moreover, epileptogenesis is modulated by Fn by modulating neuronal cell plasticity and mechanical properties in the hippocampus in epilepsy *via* its integrin receptor (Wu et al., 2017). Fn is rapidly synthesized, which was assumed by proliferated astrocytes in the hippocampus of epileptic rats (Niquet et al., 1994; Hoffman et al., 1998), and Fn and integrin interactions modulate cell adhesion and membrane elasticity in epilepsy model mice (Wu et al., 2016). Some proliferated astrocytes produce vitronectin in the hippocampus, which is related with neuronal degeneration in rat models of kainic acid (KA)-induced seizures (Niquet et al., 1996). Taken together, these findings suggest that several molecules, especially ECM molecules, contribute to reorganizing the neural network for modulating excitotoxicity in seizures in the hippocampus in lithium-pilocarpine induced SE animal models.

Lack of mitochondrial intermediate peptidase (MIP) causes seizures (Eldomery et al., 2016). Tumor necrosis factor-α receptor-associated factor 6 (TRAF6) is a key element of the transforming growth factor beta (TGFβ)-associated inflammation pathway and activates TGFβ-activated kinase 1 (TAK1) (Takaesu et al., 2000); this further leads to promoting the expression of proinflammatory cytokines and to the aggravation of inflammation (Onodera et al., 2015). Moreover, molecule causing demyelination/hypomyelination, such as gap junction alpha-1 protein (GJA1), decreases in the hippocampus on day 1 in the epileptic rat; this shows that demyelination is present in the early phases of seizure development, which is in line with the findings of our previously published study (Hobson and Garbern, 2012; Yalcinkaya et al., 2012; Basu and Sarma, 2018; Li T. et al., 2020; Wang et al., 2021). The deficit of adenosine triphosphate (ATP)-sensitive inward rectifier potassium channel 10 (KCNJ10) causes seizures and myelin vacuolization (Phani et al., 2014; Larson et al., 2018; Zhu et al., 2020). Metallothionein-1 (MT1), a zinc binding protein, exerts neuroprotection by reducing proinflammatory responses, increasing neurotrophins, and delaying neuron degeneration (Penkowa et al., 2005). In MT1-deficient mice, seizures are enhanced and neurons in hippocampus are injured, leading to apoptosis (Carrasco et al., 2000). Moreover, higher hemopexin levels are detected in the serum of schizophrenic patients than in normal subjects (Clarke et al., 1970).

Signal transducer and activator of transcription 3 (STAT3) is highly expressed in children with epilepsy (Li Y. Z. et al., 2020). Moreover, SE induced by pilocarpine activates the Janus kinase-signal transducer and activator of transcription (JAK/STAT) pathway and the STAT3-mediated signaling pathway and promotes neuronal cell death and glia activation by producing interleukin 1 beta (IL-1β) in mice with induced SE (Tian et al., 2017; Han et al., 2020). Further, inhibition of STAT3 decreases spontaneous seizure frequency and the severity of chronic epilepsy (Grabenstatter et al., 2014). DnaJ heat shock protein family member B5 (DNAJB5) and heat shock proteins (HSPs) (HSPH1 and HSPB8) are upregulated in the hippocampus of epileptic mice to protect neurons (Lee et al., 2021).

HMOX1 is upregulated in epileptic rats (Wang et al., 2013; Prakash et al., 2019), and previous studies have found that tissue-type transglutaminase (TGM2) is mostly produced by neurons in the mammalian nervous system and is elevated in neurodegenerative diseases as well as in response to acute CNS injury, which possibly induces neuronal cell death (Tucholski et al., 2006). Cathepsin S (CTSS) is mainly produced by microglia in the hippocampi of kainate-injected mice (Akahoshi et al., 2007), and prohibition of its function resulted in reducing inflammation and alleviating brain edema in a mouse model of traumatic brain injury (Xu et al., 2013). Proliferation cell nuclear antigen (PCNA) is highly expressed in epileptic animal models and in the human brain (Zhang et al., 2005; Liu et al., 2008). BDNF and the tropomyosin kinase receptor B (TRKB) pathway are predicted to function in the prevention or suppression of epilepsy targets (Lin et al., 2020; Sullivan and Kadam, 2021). The neurosecretory vascular growth factor (VGF) protein plays a critical role in the control of energy homeostasis, and the high expression of VGF in the CNS in seizure animal models is in line with a high requirement for energy (Salton et al., 2000). In addition, dystrophin is a component of gamma-aminobutyric acid (GABA)ergic synapses and plays a role in normal cognitive (i.e., episodic memory) processes (Knuesel et al., 2001; Hoogland et al., 2019); the absence of dystrophin is associated with epilepsy (Hoogland et al., 2019). Taken together, these findings provide a comprehensive picture of relevant pathways occurring during seizure development.

In conclusion, to the best of our knowledge, this study is to investigate global protein expression in the acute phase of epileptic seizures from lithium-pilocarpine induced rats using a tandem mass tag (TMT)-based proteomic approach and identified 6,157 differentially expressed proteins in total and 5,593 proteins quantified in the experimental and control groups. Of note, the majority of the differentially expressed proteins were predicted to be upregulated in the hippocampus on days 1 and 3 following lithium-pilocarpine administration, indicating that protein expression was likely to be enhanced rather than prohibited in the modulation of seizures within SE. Moreover, the number of enhanced proteins in the hippocampus decreased by more than half from day 1 to day 3, and only a small portion of proteins were the same when comparing day 1 to day 3, suggesting that different molecules and pathways are involved in epilepsy events occurring from day 1 to day 3 following lithium-pilocarpine administration. On day 1 following lithium-pilocarpine administration, as compared with controls, 10 proteins were found to be clustered in the signaling pathway in cancer (regulating sustained angiogenesis and evading apoptosis); nine proteins were upregulated, indicating the cell death processes for further seizure events. Moreover, nine upregulated proteins were predicted in complement and coagulation cascades, which participate in inflammation response, cell lysis, and phagocytosis, and four proteins were mapped to pathways relevant to MicroRNAs in cancer. On day 3 following lithium-pilocarpine administration (compared with controls), the only indicated pathways were those relevant to Huntington’s disease, TNF signaling, NF-kappa B signaling, etc., however, there were no more than four proteins in each pathway. On day 3 following lithium-pilocarpine administration (as compared with day 1), the majority of proteins were found to participate in complement and coagulation cascades, pathways relevant to cancer, and platelet activation. Our results suggest that the different molecules and pathways are involved in seizure events occurring from day 1 to day 3 following lithium-pilocarpine administration. These proteins may serve as candidate proteins for the development of seizure events and need to be studied further. Meanwhile, it is necessary to point out that the present study is a preliminary investigation. These differentially expressed proteins need to be further validated using other analyses, and a large-scale validation and a long-term strategy for proteomics analysis in the chronic phase of epileptic animals are also required.

## Data availability statement

The datasets presented in this study can be found in online repositories. The data presented in the study are deposited in FTP repository, the website is ftp://115.238.71.26, the account number is: ptm_ftp_0935 and the accession number is: B4y171.

## Ethics statement

This animal study was reviewed and approved by the National Institutes of Health and the Animal Welfare Committee of Ningxia Medical University (Ethics Approval Number: 2019-151, Ningxia, China).

## Author contributions

PW and LY conceived and designed the experiments. LY, ZC, XR, and FW performed the experiments. RY, YJ, YD, and FY analyzed the data and collected the references. PW wrote the first draft. HM, TS, and PW checked and revised the draft. All authors approved the submission of this manuscript to be published.

## Footnotes

1
https://www.maxquant.org/

2
https://www.uniprot.org/

3
http://www.ebi.ac.uk/GOA/

## References

[B1] Akahoshi N. Murashima Y. L. Himi T. Ishizaki Y. Ishii I. (2007). Increased expression of the lysosomal protease cathepsin S in hippocampal microglia following kainate-induced seizures. *Neurosci. Lett.* 429 136–141. 10.1016/j.neulet.2007.10.007 17997037

[B2] André V. Dubé C. Francóis J. Leroy C. Rigoulot M. A. Roch C. (2007). Pathogenesis and pharmacology of epilepsy in the lithium–pilocarpine model. *Epilepsia* 48 41–47. 10.1111/j.1528-1167.2007.01288.x 17910580

[B3] Basu R. Sarma J. D. (2018). Connexin 43/47 channels are important for astrocyte/oligodendrocyte cross-talk in myelination and demyelination. *J. Biosci.* 43 1055–1068. 10.1007/s12038-018-9811-0 30541963 PMC7091171

[B4] Begcevic I. Kosanam H. Martínez-Morillo E. Dimitromanolakis A. Diamandis P. Kuzmanov U. (2013). Semiquantitative proteomic analysis of human hippocampal tissues from Alzheimer’s disease and age-matched control brains. *Clin. Proteomics* 10:5. 10.1186/1559-0275-10-5 23635041 PMC3648498

[B5] Buckmaster P. S. Zhang G. F. Yamawaki R. (2002). Axon sprouting in a model of temporal lobe epilepsy creates a predominantly excitatory feedback circuit. *J. Neurosci.* 22 6650–6658. 10.1523/JNEUROSCI.22-15-06650.2002 12151544 PMC6758164

[B6] Cai D. Deng K. Mellado W. Lee J. Ratan R. R. Filbin M. T. (2002). Arginase I and polyamines act downstream from cyclic AMP in overcoming inhibition of axonal growth MAG and myelin in vitro. *Neuron* 35 711–719. 10.1016/s0896-6273(02)00826-7 12194870

[B7] Carrasco J. Penkowa M. Hadberg H. Molinero A. Hidalgo J. (2000). Enhanced seizures and hippocampal neurodegeneration following kainic acid-induced seizures in metallothionein-I + II-deficient mice. *Eur. J. Neurosci.* 12 2311–2322. 10.1046/j.1460-9568.2000.00128.x 10947810

[B8] Cavalheiro E. A. Leite J. P. Bortolotto Z. A. Turski W. A. Ikonomidou C. Turski L. (1991). Long-term effects of pilocarpine in rats: Structural damage of the brain triggers kindling and spontaneous recurrent seizures. *Epilepsia* 32 778–782. 1743148 10.1111/j.1528-1157.1991.tb05533.x

[B9] Chakir A. Fabene P. F. Ouazzani R. Bentivoglio M. (2006). Drug resistance and hippocampal damage after delayed treatment of pilocarpine-induced epilepsy in the rat. *Brain Res. Bull.* 71 127–138. 17113938 10.1016/j.brainresbull.2006.08.009

[B10] Chen Z. P. Yan Y. P. Ding Q. J. Knapp S. Potenza J. A. Schugar H. J. (1996). Effects of inhibitors of deoxyhypusine synthase on the differentiation of mouse neuroblastoma and erythroleukemia cells. *Cancer Lett.* 105 233–239. 10.1016/0304-3835(96)04287-5 8697449

[B11] Clarke H. G. Freeman T. Pryse-Phillips W. (1970). Quantitative immunoelectrophoresis of serum from hospitalized chronic schizophrenic and epileptic patients. *J. Neurol. Neurosurg. Psychiatry* 33 694–697. 10.1136/jnnp.33.5.694 5478952 PMC493551

[B12] Clifford D. B. Olney J. W. Maniotis A. Collins R. C. Zorumski C. F. (1987). The functional anatomy and pathology of lithium-pilocarpine and high-dose pilocarpine seizures. *Neuroscience* 23 953–968. 3437996 10.1016/0306-4522(87)90171-0

[B13] Dityatev A. Fellin T. (2008). Extracellular matrix in plasticity and epileptogenesis. *Neuron Glia Biol.* 4 235–247. 19497143 10.1017/S1740925X09000118

[B14] Edgar P. F. Schonberger S. J. Dean B. Faull R. L. Kydd R. Cooper G. J. (1999b). A comparative proteome analysis of hippocampal tissue from schizophrenic and Alzheimer’s disease individuals. *Mol. Psychiatry* 4 173–178. 10.1038/sj.mp.4000463 10208449

[B15] Eldomery M. K. Akdemir Z. C. Vögtle F. N. Charng W. L. Mulica P. Rosenfeld J. A. (2016). MIPEP recessive variants cause a syndrome of left ventricular non-compaction, hypotonia, and infantile death. *Genome Med.* 8:106. 10.1186/s13073-016-0360-6 27799064 PMC5088683

[B16] Fisher R. S. van Emde Boas W. Blume W. Elger C. Genton P. Lee P. (2005). Epileptic seizures and epilepsy: Definitions proposed by the international league against epilepsy (ILAE) and the international bureau for epilepsy (IBE). *Epilepsia* 46 470–472. 15816939 10.1111/j.0013-9580.2005.66104.x

[B17] Gall C. M. Lynch G. (2004). Integrins, synaptic plasticity and epileptogenesis. *Adv. Exp. Med. Biol.* 548 12–33. 15250583 10.1007/978-1-4757-6376-8_2

[B18] Ganapathi M. Padgett L. R. Yamada K. Devinsky O. Willaert R. Person R. (2019). Recessive rare variants in deoxyhypusine synthase, an enzyme involved in the synthesis of hypusine, are associated with a neurodevelopmental disorder. *Am. J. Hum. Genet.* 104 287–298. 10.1016/j.ajhg.2018.12.017 30661771 PMC6369575

[B19] Glien M. Brandt C. Potschka H. Löscher W. (2002). Effects of the novel antiepileptic drug levetiracetam on spontaneous recurrent seizures in the rat pilocarpine model of temporal lobe epilepsy. *Epilepsia* 43 350–357. 11952764 10.1046/j.1528-1157.2002.18101.x

[B20] Grabenstatter H. L. Del Angel Y. C. Carlsen J. Wempe M. F. White A. M. Cogswell M. (2014). The effect of STAT3 inhibition on status epilepticus and subsequent spontaneous seizures in the pilocarpine model of acquired epilepsy. *Neurobiol. Dis.* 62 73–85. 10.1016/j.nbd.2013.09.003 24051278 PMC3908775

[B21] Hamilton S. E. Loose M. D. Qi M. Levey A. I. Hille B. McKnight G. S. (1997). Disruption of the m1 receptor gene ablates muscarinic receptor-dependent M current regulation and seizure activity in mice. *Proc. Natl. Acad. Sci. U.S.A.* 94 13311–13316. 9371842 10.1073/pnas.94.24.13311 PMC24305

[B22] Han C. L. Liu Y. P. Guo C. J. Du T. T. Jiang Y. Wang K. L. (2020). The lncRNA H19 binding to let-7b promotes hippocampal glial cell activation and epileptic seizures by targeting Stat3 in a rat model of temporal lobe epilepsy. *Cell Prolif.* 53:e12856. 10.1111/cpr.12856 32648622 PMC7445408

[B23] Hobson G. M. Garbern J. Y. (2012). Pelizaeus-Merzbacher disease. Pelizaeus-merzbacher-like disease 1, and related hypomyelinating disorders. *Semin. Neurol.* 32 62–67. 10.1055/s-0032-1306388 22422208

[B24] Hoffman K. B. Pinkstaff J. K. Gall C. M. Lynch G. (1998). Seizure induced synthesis of fibronectin is rapid and age dependent: Implications for long-term potentiation and sprouting. *Brain Res.* 812 209–215. 9813331 10.1016/s0006-8993(98)00727-6

[B25] Honchar M. P. Olney J. W. Sherman W. R. (1983). Systemic cholinergic agents induce seizures and brain damage in lithium-treated rats. *Science* 220, 323–325. 10.1126/science.6301005. 6301005

[B26] Hondius D. C. van Nierop P. Li K. W. Hoozemans J. J. M. van der Schors R. C. van Haastert E. S. (2016). Profiling the human hippocampal proteome at all pathologic stages of Alzheimer’s disease. *Alzheimers Dement*. 12 654–668. 10.1016/j.jalz.2015.11.002 26772638

[B27] Hoogland G. Hendriksen R. G. F. Slegers R. J. Hendriks M. P. H. Schijns O. E. M. G. Aalbers M. W. (2019). The expression of the distal dystrophin isoforms Dp140 and Dp71 in the human epileptic hippocampus in relation to cognitive functioning. *Hippocampus* 29 102–110. 10.1002/hipo.23015 30069964

[B28] Huang Y. Higginson D. S. Hester L. Park M. H. Snyder S. H. (2007). Neuronal growth and survival mediated by eIF5A, a polyamine-modified translation initiation factor. *Proc. Natl. Acad. Sci. U.S.A.* 104 4194–4199. 10.1073/pnas.0611609104 17360499 PMC1820731

[B29] Kim S. Burette A. Chung H. S. Kwon S. K. Woo J. Lee H. W. (2006). NGL family PSD-95-interacting adhesion molecules regulate excitatory synapse formation. *Nat. Neurosci.* 9 1294–1301. 16980967 10.1038/nn1763

[B30] Knuesel I. Zuellig R. A. Schaub M. C. Fritschy J. M. (2001). Alterations in dystrophin and utrophin expression parallel the reorganization of GABAergic synapses in a mouse model of temporal lobe epilepsy. *Eur. J. Neurosci.* 13 1113–1124. 10.1046/j.0953-816x.2001.01476.x 11285009

[B31] Larson V. A. Mironova Y. Vanderpool K. G. Waisman A. Rash J. E. Agarwal A. (2018). Oligodendrocytes control potassium accumulation in white matter and seizure susceptibility. *eLife.* 7:e34829. 10.7554/eLife.34829 29596047 PMC5903864

[B32] Lee T. S. Li A. Y. Rapuano A. Mantis J. Eid T. Seyfried T. N. (2021). Gene expression in the epileptic (EL) mouse hippocampus. *Neurobiol. Dis.* 147:105152. 10.1016/j.nbd.2020.105152 33153970

[B33] Leite J. P. Bortolotto Z. A. Cavalheiro E. A. (1990). Spontaneous recurrent seizures in rats: An experimental model of partial epilepsy. *Neurosci. Biobehav. Rev.* 14 511–517. 2287490 10.1016/s0149-7634(05)80076-4

[B34] Li A. L. Li H. Y. Jin B. F. Ye Q. N. Zhou T. Yu X. D. (2004). A novel eIF5A complex functions as a regulator of p53 and p53-dependent apoptosis. *J. Biol. Chem.* 279 49251–49258. 10.1074/jbc.M407165200 15371445

[B35] Li T. Niu J. Yu G. Ezan P. Yi C. Wang X. (2020). Connexin 43 deletion in astrocytes promotes CNS remyelination by modulating local inflammation. *Glia* 68 1201–1212. 10.1002/glia.23770 31868275

[B36] Li Y. Z. Zhang L. Liu Q. Bian H. T. Cheng W. J. (2020). The effect of single nucleotide polymorphisms of STAT3 on epilepsy in children. *Eur. Rev. Med. Pharmacol. Sci.* 24 837–842. 10.26355/eurrev_202001_20067 32016989

[B37] Lin J. C. Ho W. H. Gurney A. Rosenthal A. (2003). The netrin-G1 ligand NGL-1 promotes the outgrowth of thalamocortical axons. *Nat. Neurosci.* 6 1270–1276. 14595443 10.1038/nn1148

[B38] Lin T. W. Harward S. C. Huang Y. Z. McNamara J. O. (2020). Targeting BDNF/TrkB pathways for preventing or suppressing epilepsy. *Neuropharmacology* 167:107734. 10.1016/j.neuropharm.2019.107734 31377199 PMC7714524

[B39] Liu Y. W. Curtis M. A. Gibbons H. M. Mee E. W. Bergin P. S. Teoh H. H. (2008). Doublecortin expression in the normal and epileptic adult human brain. *Eur. J. Neurosci.* 28 2254–2265. 10.1111/j.1460-9568.2008.06518.x 19046368

[B40] Niquet J. Gillian A. Ben-Ari Y. Represa A. (1996). Reactive glial cells express a vitronectin-like protein in the hippocampus of epileptic rats. *Glia* 16 359–367. 10.1002/(SICI)1098-1136(199604)16:4<359:AID-GLIA8<3.0.CO;2-V 8721676

[B41] Niquet J. Jorquera I. Ben-Ari Y. Represa A. (1994). Proliferative astrocytes may express fibronectin-like protein in the hippocampus of epileptic rats. *Neurosci. Lett.* 180 13–16. 10.1016/0304-3940(94)90902-4 7877752

[B42] Onodera Y. Teramura T. Takehara T. Shigi K. Fukuda K. (2015). Reactive oxygen species induce Cox-2 expression *via* TAK1 activation in synovial fibroblast cells. *FEBS Open Bio.* 5 492–501. 10.1016/j.fob.2015.06.001 26110105 PMC4476901

[B43] Pan Y. Liu G. Fang M. Shen L. Wang L. Han Y. (2010). Abnormal expression of netrin-G2 in temporal lobe epilepsy neurons in humans and a rat model. *Exp. Neurol.* 224 340–346. 10.1016/j.expneurol.2010.04.001 20382146

[B44] Park M. H. Kar R. K. Banka S. Ziegler A. Chung W. K. (2022). Post-translational formation of hypusine in eIF5A: Implications in human neurodevelopment. *Amino Acids* 54 485–499. 10.1007/s00726-021-03023-6 34273022 PMC9117371

[B45] Penkowa M. Florit S. Giralt M. Quintana A. Molinero A. Carrasco J. (2005). Metallothionein reduces central nervous system inflammation, neurodegeneration, and cell death following kainic acid-induced epileptic seizures. *J. Neurosci. Res.* 79 522–534. 10.1002/jnr.20387 15614785

[B46] Persike D. S. Marques-Carneiro J. E. Stein M. L. D. L. Yacubian E. M. T. Centeno R. Canzian M. (2018). Altered proteins in the hippocampus of patients with mesial temporal lobe epilepsy. *Pharmaceuticals (Basel).* 11:E95. 10.3390/ph11040095 30274397 PMC6316307

[B47] Persike D. Lima M. Amorim R. Cavalheiro E. Yacubian E. Centeno R. (2012). Hippocampal proteomic profile in temporal lobe epilepsy. *J. Epilepsy Clin. Neurophysiol*. 18 53–56. 10.1590/S1676-26492012000200007

[B48] Phani N. M. Acharya S. Xavy S. Bhaskaranand N. Bhat M. K. Jain A. (2014). Genetic association of KCNJ10 rs1130183 with seizure susceptibility and computational analysis of deleterious non-synonymous SNPs of KCNJ10 gene. *Gene* 536 247–253. 10.1016/j.gene.2013.12.026 24378235

[B49] Pitkanen A. Ndode-Ekane X. E. Lukasiuk K. Wilczynski G. M. Dityatev A. Walker M. C. (2014). Neural ECM and epilepsy. *Prog. Brain Res.* 214 229–262. 25410361 10.1016/B978-0-444-63486-3.00011-6

[B50] Prakash C. Mishra M. Kumar P. Kumar V. Sharma D. (2019). Dehydroepiandrosterone alleviates oxidative stress and apoptosis in iron-induced epilepsy *via* activation of Nrf2/ARE signal pathway. *Brain Res. Bull.* 153 181–190. 10.1016/j.brainresbull.2019.08.019 31472186

[B51] Priel M. R. Albuquerque E. X. (2002). Short-term effects of pilocarpine on rat hippocampal neurons in culture. *Epilepsia* 43 40–46. 10.1046/j.1528-1157.43.s.5.18.x 12121294

[B52] Racine R. J. (1972). Modification of seizure activity by electrical stimulation. II. Motor seizure. *Electroencephalogr. Clin. Neurophysiol.* 32 281–294. 10.1016/0013-4694(72)90177-0 4110397

[B53] Sadeghi L. Rizvanov A. A. Dabirmanesh B. Salafutdinov I. I. Sayyah M. Shojaei A. (2021). Proteomic profiling of the rat hippocampus from the kindling and pilocarpine models of epilepsy: Potential targets in calcium regulatory network. *Sci. Rep*. 11:8252. 10.1038/s41598-021-87555-7 33859251 PMC8050094

[B54] Salton S. R. Ferri G. L. Hahm S. Snyder S. E. Wilson A. J. Possenti R. (2000). VGF: A novel role for this neuronal and neuroendocrine polypeptide in the regulation of energy balance. *Front. Neuroendocrinol.* 21:199–219. 10.1006/frne.2000.0199 10882540

[B55] Schuller A. P. Wu C. C. Dever T. E. Buskirk A. R. Green R. (2017). eIF5A functions globally in translation elongation and termination. *Mol. Cell* 66 194.e–205.e. 28392174 10.1016/j.molcel.2017.03.003 PMC5414311

[B56] Sullivan B. J. Kadam S. D. (2021). Brain-derived neurotrophic factor in neonatal seizures. *Pediatr. Neurol.* 118 35–39. 10.1016/j.pediatrneurol.2021.01.011 33773288 PMC8076080

[B57] Sultana R. Boyd-Kimball D. Cai J. Pierce W. M. Klein J. B. Merchant M. (2007). Proteomics analysis of the Alzheimer’s disease hippocampal proteome. *J. Alzheimers Dis*. 11 153–164. 10.3233/jad-2007-11203 17522440

[B58] Takaesu G. Kishida S. Hiyama A. Yamaguchi K. Shibuya H. Irie K. (2000). TAB2, a novel adaptor protein, mediates activation of TAK1 MAPKKK by linking TAK1 to TRAF6 in the IL-1 signal transduction pathway. *Mol. Cell* 5 649–658. 10.1016/s1097-2765(00)80244-0 10882101

[B59] Tian D. S. Peng J. Murugan M. Feng L. J. Liu J. L. Eyo U. B. (2017). Chemokine CCL2-CCR2 signaling induces neuronal cell death *via* STAT3 activation and IL-1β production after status epilepticus. *J. Neurosci.* 37 7878–7892. 10.1523/JNEUROSCI.0315-17.2017 28716963 PMC5559763

[B60] Tucholski J. Roth K. A. Johnson G. V. (2006). Tissue transglutaminase overexpression in the brain potentiates calcium-induced hippocampal damage. *J. Neurochem.* 97 582–594. 10.1111/j.1471-4159.2006.03780.x 16539654

[B61] Turski W. A. Cavalheiro E. A. Schwarz M. Czuczwar S. L. J. Kleinrok Z. Turski L. (1983a). Limbic seizures produced by pilocarpine in rats: Behavioural, electroencephalographic and neuropathological study. *Behav. Brain Res.* 9 315–335. 6639740 10.1016/0166-4328(83)90136-5

[B62] Turski W. A. Czuczwar S. J. Kleinrok Z. Turski L. (1983b). Cholinomimetics produce seizures and brain damage in rats. *Experientia* 39 1408–1411. 6140182 10.1007/BF01990130

[B63] Wang P. Ma K. Yang L. Zhang G. Ye M. Wang S. (2021). Predicting signaling pathways regulating demyelination in a rat model of lithium-pilocarpine-induced acute epilepsy: A proteomics study. *Int. J. Biol. Macromol.* 193 1457–1470. 10.1016/j.ijbiomac.2021.10.209 34742844

[B64] Wang W. Wang W. P. Zhang G. L. Wu Y. F. Xie T. Kan M. C. (2013). Activation of Nrf2-ARE signal pathway in hippocampus of amygdala kindling rats. *Neurosci. Lett.* 543 58–63. 10.1016/j.neulet.2013.03.038 23570726

[B65] Woo J. Kwon S. K. Kim E. (2009). The NGL family of leucine-rich repeat-containing synaptic adhesion molecules. *Mol. Cell. Neurosci.* 42 1–10. 19467332 10.1016/j.mcn.2009.05.008

[B66] Wu X. Reddy D. S. (2012). Integrins as receptor targets for neurological disorders. *Pharmacol. Ther.* 134 68–81. 22233753 10.1016/j.pharmthera.2011.12.008 PMC3288359

[B67] Wu X. Muthuchamy M. Reddy D. S. (2016). Atomic force microscopy protocol for measurement of membrane plasticity and extracellular interactions in single neurons in epilepsy. *Front. Aging Neurosci.* 8:88. 10.3389/fnagi.2016.00088 27199735 PMC4854888

[B68] Wu X. Muthuchamy M. Reddy D. S. (2017). Atomic force microscopy investigations of fibronectin and α5β1-integrin signaling in neuroplasticity and seizure susceptibility in experimental epilepsy. *Epilepsy Res.* 138 71–80. 10.1016/j.eplepsyres.2017.10.013 29096132

[B69] Xu J. Wang H. Ding K. Lu X. Li T. Wang J. (2013). Inhibition of cathepsin S produces neuroprotective effects after traumatic brain injury in mice. *Mediators Inflamm.* 2013:187873. 10.1155/2013/187873 24282339 PMC3824312

[B70] Yalcinkaya C. Erturk O. Tuysuz B. Yesil G. Verbeke J. I. Keyser B. (2012). A novel GJC2 mutation associated with hypomyelination and Müllerian agenesis syndrome: Coincidence or a new entity? *Neuropediatrics* 43 159–161. 10.1055/s-0032-1313912 22610664

[B71] Yang J. W. Czech T. Lubec G. (2004). Proteomic profiling of human hippocampus. *Electrophoresis* 25 1169–1174. 10.1002/elps.200305809 15095461

[B72] Zhang S. Zhu C. Liu Q. Wang W. (2005). Effects of chloroquine on GFAP. PCNA and cyclin D1 in hippocampus and cerebral cortex of rats with seizures induced by pentylenetetrazole. *J. Huazhong Univ. Sci. Technolog. Med. Sci.* 25 625–628. 10.1007/BF02896153 16696308

[B73] Zhu H. Zhang M. Fu Y. Long H. Xiao W. Feng L. (2020). Effects of AQP4 and KCNJ10 gene polymorphisms on drug resistance and seizure susceptibility in Chinese Han patients with focal epilepsy. *Neuropsychiatr. Dis. Treat.* 16 119–129. 10.2147/NDT.S231352 32021205 PMC6957104

